# Immune Factors Linked to Long-Term HCV Humoral Memory Five Years After Cure in People with HIV: A Cross-Sectional Study

**DOI:** 10.3390/ph19060854

**Published:** 2026-05-29

**Authors:** Rafael Amigot-Sánchez, Daniel Sepúlveda-Crespo, Rubén Martin Escolano, Laura Tarancon-Diez, Ana Virseda-Berdices, Juan Berenguer, Juan González-García, Cristina Diez, Víctor Hontañón, Belén Yélamos, Julián Gómez, Elena Vázquez-Alejo, José Luis Jimenez, María A. Jiménez-Sousa, Isidoro Martínez, Salvador Resino

**Affiliations:** 1 Unidad de Infección Viral e Inmunidad, Centro Nacional de Microbiología (CNM), Instituto de Salud Carlos III (ISCIII), 28220 Madrid, Spain; rafael.amigot@isciii.es (R.A.-S.);; 2 Centro de Investigación Biomédica en Red en Enfermedades Infecciosas (CIBERINFEC), Instituto de Salud Carlos III (ISCIII), 28220 Madrid, Spain; 3 Escuela de Doctorado de Microbiología, Universidad Autónoma de Madrid, 28049 Madrid, Spain; 4 Grupo de Infecciones en la Población Pediátrica, Instituto de Investigación Sanitaria del Gregorio Marañón (IiSGM), 28007 Madrid, Spain; 5 Unidad de Enfermedades Infecciosas/VIH, Hospital General Universitario Gregorio Marañón, 28007 Madrid, Spain; 6 Instituto de Investigación Sanitaria del Gregorio Marañón (IiSGM), 28007 Madrid, Spain; 7 Unidad de VIH, Servicio de Medicina Interna, Hospital Universitario “La Paz”, 28046 Madrid, Spain; 8 Departamento de Bioquímica y Biología Molecular, Facultad de Ciencias Químicas, Universidad Complutense, 28040 Madrid, Spain; mbyelamos@quim.ucm.es (B.Y.); jgomezgu@ucm.es (J.G.); 9 Laboratorio de Inmunobiología Molecular, Hospital General Universitario Gregorio Marañón, 28007 Madrid, Spain; 10 Centro de Investigación Biomédica en Red de Bioingeniería, Biomateriales y Nanomedicina (CIBER-BBN), Instituto de Salud Carlos III (ISCIII), 28220 Madrid, Spain; 11 Spanish HIV-HGM BioBank, 28007 Madrid, Spain

**Keywords:** HCV/HIV coinfection, sustained virologic response, HCV neutralizing antibodies, T-cell exhaustion, immunological scar, biomarkers

## Abstract

**Background:** The immunological factors associated with long-term hepatitis C virus (HCV)-specific humoral immunity after cure remain uncharacterized, particularly in people with HIV (PWH). This study investigated T-cell immunophenotypes and plasma biomarkers associated with anti-E2 binding (HCV-E2Abs) and neutralizing antibody (HCV-nAbs) titers 5 years after achieving sustained virologic response (SVR). **Methods:** This cross-sectional study analyzed 64 PWH with cured HCV and prior advanced fibrosis. We quantified plasma antibody titers against 5 HCV genotypes, T-cell phenotypes (n = 58), and plasma biomarkers (n = 50). Associations were assessed using Generalized Linear Models (gamma distribution, log-link function) adjusted for clinical confounders, reporting adjusted Arithmetic Mean Ratios (aAMRs) and false discovery rate (FDR)-corrected q-values. **Results:** Higher frequencies of CD4^+^ T-cell activation (CD38^+^; aAMR = 1.58; q = 0.028) and soluble CD27 levels (aAMR = 1.46; q = 0.038) were associated with higher HCV-E2Abs titers. In contrast, memory T-cell activation across CD4^+^ and CD8^+^ compartments (HLA-DR^+^ and CD38+; all q < 0.10) and elevated soluble immune checkpoints (sCD28, sPD-L2, sLAG-3, sCTLA-4; all q < 0.10) were associated with preserved HCV-nAbs titers. Conversely, a higher frequency of naïve CD8^+^ T-cells was associated with lower neutralization capacity (aAMR = 0.41; q = 0.042). Regarding inflammatory markers, soluble TNF-RI was positively associated with neutralizing titers (aAMR = 1.44; q = 0.019), whereas IL-18 was inversely associated (aAMR = 0.53; q = 0.019). **Conclusions:** Specific activated T-cell subsets, checkpoint shedding, and selective inflammatory signals were associated with higher long-term HCV-nAbs titers in PWH. In contrast, higher frequencies of naïve CD8^+^ T-cells and elevated IL-18 levels were associated with reduced neutralizing capacity.

## 1. Introduction

Antiviral therapy against hepatitis C virus (HCV) achieves sustained virologic response (SVR) rates exceeding 95%, regardless of HIV status [1,2]. However, viral clearance does not guarantee immediate immunological restoration. In people with HIV (PWH) with a history of advanced liver fibrosis, a persistent “immunological scar”, characterized by chronic T-cell activation, exhaustion, and altered lymphoid architecture, often endures for years after HCV cure [3,4,5].

The long-term durability of HCV-specific humoral memory in the post-SVR era remains incompletely understood. While anti-HCV antibodies naturally decline following antigen removal, the rate and quality of this decay are heterogeneous [6,7]. This variability is especially relevant for PWH, who remain at increased risk of HCV reinfection and in whom the loss of the primary B-cell stimulus is compounded by HIV-associated CD4^+^ T-cell dysfunction [8,9]. To date, it remains unknown whether the immunological determinants governing the magnitude of binding antibodies (quantity) differ from those sustaining neutralizing capacity (quality).

Beyond cellular subsets, the systemic environment regulates humoral memory [10,11,12,13,14]. Soluble immune checkpoints (ICPs) and inflammatory mediators serve as indicators of bone marrow niche stability, where long-lived plasma cells (LLPCs) reside to sustain humoral memory [15,16,17]. While lymphocyte turnover markers may indicate functional immune activity [18], innate inflammation or inhibitory signaling could disrupt these survival niches [15,17,19]. Characterizing how distinct T-cell differentiation states and soluble immune factors relate to antibody persistence is essential for identifying benchmarks of durable immunity [20].

It remains unresolved whether higher long-term antibody titers following HCV cure are associated with the restoration of immune homeostasis or with residual chronic activation. While natural infection does not confer sterilizing immunity, persistent neutralizing capacity may nonetheless serve as a biomarker for functional adaptive memory and modulate reinfection kinetics. In PWH, these antibody profiles reflect the systemic immunological milieu, where residual perturbations persist. We therefore characterized the specific cellular and soluble factors associated with HCV-specific binding and neutralizing antibody titers five years after SVR in a cross-sectional analysis of PWH with prior advanced fibrosis.

## 2. Results

### 2.1. Baseline Characteristics

The study population comprised 64 HIV/HCV-coinfected individuals with a history of advanced liver fibrosis (Table 1). The final analytical cohorts included 58 participants for T-cell immunophenotyping and 50 for plasma biomarker quantification (Supplementary Table S4). The cohort was predominantly male (80%), with a median age of 51 years and a high prevalence of prior intravenous drug use (77%). Participants had a median CD4^+^ count of 486 cells/mm^3^ and a history of immunosuppression (median nadir CD4^+^: 130 cells/mm^3^). Over half of the patients (53%) were HCV treatment-experienced, primarily with interferon-based regimens. Regarding liver status, advanced disease was confirmed by a median LSM of 21.2 kPa, with 45% of the cohort presenting an LSM ≥ 25 kPa, indicative of compensated cirrhosis.

### 2.2. CD4^+^ T-Cell Immunophenotyping and Long-Term HCV Humoral Memory

Adjusted models identified specific T-cell signatures associated with antibody outcomes (Table 2; unadjusted data in Supplementary Table S5).

Elevated frequencies of activated CD38^+^ cells within the total CD4^+^ population (aAMR = 1.58; q = 0.028) and CD38^+^HLA-DR^+^ cells (aAMR = 1.24; q = 0.037) were associated with higher HCV-E2Abs titers. Higher frequencies of CD38^+^ cells within the naïve (CD38^+^: aAMR = 1.33; q = 0.003), central memory (CD38^+^: aAMR = 1.31; q = 0.034), and TemRA compartments (CD38^+^: aAMR = 1.24; q = 0.034) were associated with higher HCV-E2Abs titers. Similarly, higher frequencies of CD38^+^HLA-DR^+^ cells within the naïve (aAMR = 1.13; q = 0.034) and TemRA (aAMR = 1.15; q = 0.052) subsets were associated with higher HCV-E2Abs titers.

Within the total CD4^+^ population, higher frequencies of CD38^+^ (aAMR = 2.05; q = 0.079) and CD57^+^ (aAMR = 1.54; q = 0.087) cells were associated with higher HCV-nAbs titers. Higher frequencies of activated cells within the naïve (CD38^+^: aAMR = 2.29; q = 0.003; CD38^+^HLA-DR^+^: aAMR = 1.42; q = 0.073) and central memory subsets (CD38^+^: aAMR = 2.14; q = 0.073; HLA-DR^+^: aAMR = 1.70; q = 0.099; CD38^+^HLA-DR^+^: aAMR = 1.79; q = 0.073) were associated with higher HCV-nAbs titers. Conversely, higher HLA-DR^+^ expression within the effector memory compartment was inversely associated with HCV-nAbs titers (aAMR = 0.59; q = 0.090). Finally, within the TemRA subset, higher frequencies of CD38^+^ (aAMR = 1.55; q = 0.087), CD57^+^ (aAMR = 1.49; q = 0.087), and CD127^+^ (aAMR = 1.58; q = 0.014) cells were associated with higher HCV-nAbs titers.

### 2.3. CD8^+^ T-Cell Immunophenotyping and Long-Term HCV Humoral Memory

Adjusted models of the CD8^+^ compartment evaluated associations with HCV-E2Abs and HCV-nAbs titers (Table 3; unadjusted data in Supplementary Table S7). Regarding HCV-E2Abs titers, no CD8^+^ T-cell subsets were associated with the outcome after FDR correction.

For HCV-nAbs, higher frequencies of HLA-DR^+^ CD8^+^ T-cells within the central memory (aAMR = 2.17; q = 0.045), effector memory (aAMR = 1.80; q = 0.045), and TemRA (aAMR = 1.16; q = 0.078) compartments were associated with higher HCV-nAbs titers. The co-expression of CD38 and HLA-DR within the effector memory (aAMR = 1.53; q = 0.060) and TemRA (aAMR = 1.21; q = 0.045) subsets was associated with higher HCV-nAbs titers. CD38^+^ expression in TemRA (aAMR = 1.47; q = 0.003) and naïve (aAMR = 1.17; q = 0.056) cells was associated with higher HCV-nAbs titers. The frequency of effector memory CD8^+^ T-cells was positively associated with HCV-nAbs titers (aAMR = 1.57; q = 0.060), whereas the frequency of naïve CD8^+^ T-cells was inversely associated with HCV-nAbs titers (aAMR = 0.41; q = 0.042).

### 2.4. Plasma Biomarkers and Long-Term HCV Humoral Memory

Adjusted models evaluated associations between plasma biomarkers and antibody titers (Table 4; unadjusted data in Supplementary Table S9). Regarding HCV-E2Abs titers, higher sCD27 levels were associated with higher HCV-E2Abs titers (aAMR = 1.46; q = 0.038).

In contrast, higher levels of sCD28 (aAMR = 1.68; q = 0.029), sPD-L2 (aAMR = 2.50; q = 0.048), sLAG-3 (aAMR = 1.97; q = 0.063), and sCTLA-4 (aAMR = 1.46; q = 0.086) were associated with higher HCV-nAbs titers. Among inflammatory markers, soluble TNF-RI levels were positively associated with higher HCV-nAbs titers (aAMR = 1.44; q = 0.019), whereas IL-18 was inversely associated with HCV-nAbs titers (aAMR = 0.53; q = 0.019).

### 2.5. Sensitivity Analyses

Finally, to evaluate the stability of our findings across both cellular and soluble compartments, we performed a sensitivity analysis substituting the continuous LSM covariate with the historical HCV treatment regimen (IFN-based vs. DAA-based). The primary immunological dichotomy remained consistent: focal CD4^+^ activation and sCD27 were associated with binding titers, whereas CD8^+^ effector memory activation, checkpoint shedding, and IL-18 were associated with neutralizing titers (Supplementary Tables S6, S8 and S10).

## 3. Discussion

Our findings reveal distinct immunological profiles associated with the magnitude of HCV humoral memory in PWH five years after HCV cure. Focal CD4^+^ T-cell activation and elevated sCD27 turnover are associated with higher binding titers (HCV-E2Abs). Conversely, the presence of a broader “immunological scar,” characterized by central and effector memory activation in both CD4^+^ and CD8^+^ compartments, CD4^+^ senescence, and elevated soluble ICPs is associated with higher overall neutralizing capacity (HCV-nAbs). We identified a potential trade-off in immune reconstitution: a higher frequency of naïve CD8^+^ T-cells and elevated IL-18 levels were associated with lower neutralization capacity, whereas regulatory shedding (sTNF-RI) was associated with higher neutralizing titers.

### 3.1. Factors Associated with Anti-E2 Binding Antibodies

Our adjusted models demonstrated that focal CD4^+^ T-cell activation (CD38^+^) across multiple differentiation stages, as well as sCD27, was associated with HCV-E2Abs. Although a trend toward homeostatic renewal was observed (CD127^+^ naïve CD4^+^ T-cells), it did not reach statistical significance after FDR correction (*p* = 0.030; q = 0.105). This suggests that ongoing CD4^+^ T-cell turnover and basal activation, rather than broad immune exhaustion, are associated with higher circulating binding antibodies post-cure. Furthermore, sCD27 reflects lymphocyte turnover within the CD27-CD70 axis, which is critical for plasma cell survival [18,21]. Together with the CD4^+^ CD38^+^ signature, this profile suggests that the total antibody pool is maintained by ongoing, antigen-independent signals supporting short-lived plasmablasts or early LLPCs [16,22].

### 3.2. Factors Associated with Neutralizing Antibodies

Memory subset activation (HLA-DR/CD38) and senescence (CD57) emerged as factors associated with higher overall neutralizing capacity. Specifically, CD57^+^ expression within the CD4^+^ compartment and HLA-DR^+^/CD38^+^ activation across CD8^+^ memory subsets were associated with higher HCV-nAbs titers. While CD57 is classically defined as a senescence marker [23], in chronic viral infections, CD57-expressing T-cells represent terminally differentiated effectors with high cytotoxic potential and distinct migratory properties [24,25,26]. Our data indicate that this “exhausted” phenotype coexists with preserved humoral function after HCV cure. The residual inflammatory signaling maintaining this highly differentiated T-cell profile might concurrently provide an environment supporting antibody-secreting cells [27].

In PWH on ART, low-level HIV replication and microbial translocation sustain baseline immune activation [28,29]. Variations in this systemic “noise” may act as an antigen surrogate, providing tonic stimulation that sustains the exhausted memory pool. This aligns with observations where blocking inflammatory pathways accelerated the decay of established humoral memory [30]. The identified activated CD4^+^ T-cells (CD38^+^HLA-DR^+^) may overlap with circulating T follicular helper cells, which are essential for maintaining high-affinity B-cell responses [31,32]. Furthermore, the associations with activated CD8^+^ cells suggest that cytotoxic effector and B-cell cross-talk may help maintain antibody clones even in the absence of cognate antigen [33]. However, our models reveal a constraint within this activated landscape: HLA-DR^+^ expression specifically within the CD4^+^ effector memory compartment was inversely associated with neutralizing titers. In HIV pathogenesis, elevated HLA-DR on CD4^+^ T-cells is a hallmark of hyperactivation and susceptibility to activation-induced cell death [9,28,31]. This dichotomy suggests an immunological trade-off. While broad CD8^+^ activation and checkpoint shedding may support long-lived plasma cell persistence, hyperactivation of the CD4^+^ effector memory pool likely compromises the direct T-cell help required to sustain high-affinity neutralizing clones [8,34].

Alternatively, these associations may reflect a pre-cure “legacy effect”. Mechanistically, broad HCV neutralization typically develops after prolonged antigenic stimulation and viral diversification, reflecting extensive affinity maturation [35]. Consequently, high post-SVR HCV-nAbs may serve as a surrogate marker of historical antigenic burden; individuals who developed potent nAbs during chronic infection accumulated stronger exhaustion and senescence imprints. Given the cross-sectional design, we cannot fully disentangle whether these activated signatures actively maintain HCV-nAbs or reflect a shared prior immunological trajectory established during chronic infection.

The inverse association between naïve CD8^+^ T-cell frequency and HCV-nAb titers indicates that in individuals with more robust immune recovery, specific neutralizing memory is lower. This aligns with the “niche competition” theory of plasma cell survival [36,37,38]. LLPCs require specialized survival signals (e.g., APRIL, IL-6) to persist within limited bone marrow niches [13,14]. In this mode, the withdrawal of inflammatory stimuli, concomitant with naïve compartment expansion, could lead to the depletion of stromal factors, potentially resulting in the displacement or apoptosis of HCV-specific clones [39]. Thus, immune reconstitution may inadvertently be associated with a reduced neutralizing arsenal in PWH.

Alternatively, this inverse relationship may reflect a clinical trajectory effect rather than active displacement. Participants with superior naïve CD8^+^ restoration experienced lower historical immune activation and lymphoid scarring. Because broad HCV neutralization requires prolonged, antigen-driven affinity maturation [35], individuals with less severe chronic immunopathology generated weaker overall pre-cure HCV-nAb responses, resulting in lower baseline levels detected at year 5. Under this paradigm, a preserved naïve compartment serves as a biomarker of a milder chronic infection, which generated a weaker neutralizing response.

Elevated levels of soluble ICPs (sCD28, sLAG-3, sCTLA-4, and sPD-L2) were associated with higher neutralizing titers. Generated via proteolytic shedding upon T-cell activation [40,41], these soluble isoforms likely reflect residual basal activation, representing the “immunological scar,” rather than active immunosuppression. This ongoing basal activation appears necessary to sustain high-affinity clones [40,42]. While epigenetic and molecular scars constrain overall T-cell function, they do not abolish high-affinity memory [43,44]. This aligns with evidence that HCV-driven T-cell dysfunction and transcriptional perturbations are only partially reversible post-cure, particularly in PWH [3,5].

One potential mechanism to explain this association is that soluble checkpoints can act as decoy receptors, interfering with membrane-bound inhibitory receptor–ligand interactions [45,46]. In such a scenario, by buffering negative signaling, these molecules could preserve essential helper T-cell functions, thereby being associated with a higher neutralizing antibody pool within a chronically exhausted immune landscape.

The positive association of sTNF-RI with HCV-nAb titers aligns with LLPC niche biology. Because TNF-α disrupts the bone marrow plasma cell niche [17], elevated sTNF-RI, a decoy receptor buffering TNF-α bioactivity, likely reflects a compensatory mechanism protecting LLPC persistence within an activated host environment [47]. Conversely, IL-18 emerged as the sole negative factor among the inflammatory biomarkers. Unlike adaptive checkpoint shedding, this hallmark of inflammasome activation indicates that chronic innate “noise” may disrupt functional antibody maintenance [16,48], which aligns with the inverse association observed in our cohort. Biologically, IL-18 is known to drive hepatic fibrogenesis [49,50,51] and trigger pyroptosis in PWH. This creates a hostile systemic microenvironment that could compromise bone marrow stromal cells [52,53], which is consistent with the observed lower neutralizing titers.

Baseline liver disease severity is a determinant of the post-SVR immunological landscape. The magnitude of the systemic immunological scar correlates with baseline liver stiffness [54], and the post-SVR decay of inflammatory biomarkers is driven by LSM regression [55,56]. Because our cohort targeted individuals with historical advanced liver fibrosis, the observed T-cell exhaustion and checkpoint shedding signatures reflect an altered immune milieu. In PWH who achieve SVR at earlier stages of liver disease, this residual scar is likely less pronounced. Consequently, the immunological determinants of long-term humoral memory in populations with mild liver disease might be attenuated or differ from the signatures identified here.

### 3.3. Clinical and Translational Implications

The dissociation between binding magnitude and functional neutralization has clinical implications. First, it cautions against using binding titers as surrogates for protective immunity against HCV in cured PWH, as anti-E2 titers may remain detectable without concurrent neutralizing capacity. Second, the association of activated/senescent signatures with HCV-nAbs indicates that post-SVR immune “normalization” does not equate to higher functional memory.

Following viral clearance, serum neutralization often declines despite memory B-cell persistence, indicating that the bottleneck for durable immunity is LLPC maintenance [57]. This challenge is amplified in HIV/HCV coinfection, where HIV impairs CD4-dependent T-cell help, diminishing overall antibody responses [6,7]. Consequently, while preserved memory B-cells post-cure suggest that targeted boosting could successfully induce neutralizing antibodies [20,57], our data highlight a caveat: the systemic immune milieu, particularly inflammatory and regulatory pathways, will likely dictate whether these responses translate into durable circulating neutralization. These results suggest that the limited durability of previous vaccine candidates may be related to an inability to maintain these specific memory signals, highlighting the need to consider the systemic inflammatory environment in future immunotherapeutic strategies [20].

Finally, given the documented reinfection risks in this population [58,59], continued post-SVR screening remains essential for PWH. Identifying patients with lower neutralizing antibody titers, such as those with elevated IL-18 or a higher frequency of naïve CD8^+^ T-cells, could eventually help define risk profiles for future targeted prevention strategies, pending validation in larger cohorts.

### 3.4. Strengths and Limitations

A primary strength of this study is the five-year post-SVR follow-up horizon, providing a timeframe significantly longer than that of most existing cohorts. By focusing on PWH with prior advanced fibrosis, the findings remain clinically pertinent to a high-risk population prone to post-cure complications. Furthermore, the analysis of 19 plasma biomarkers and multiple T-cell subsets using independent, confounder-adjusted multivariable models identified a functional dichotomy that conventional single-marker analyses would overlook.

This study has several limitations. First, the cross-sectional design precludes causal inference or the assessment of intra-individual antibody decay kinetics; thus, the reported associations do not imply directionality. Second, while the cohort is well-characterized, the modest sample size (N = 50–58) limits statistical power. Therefore, associations with FDR-corrected q-values < 0.10 should be interpreted as exploratory, hypothesis-generating signals requiring large-scale validation. Third, as we did not phenotype antigen-specific B cells or bone marrow plasma cells, these findings reflect the systemic immunological environment supporting humoral memory rather than a direct assessment of the antibody-producing machinery. Fourth, we could not evaluate the independent immunological impact of HCV treatment regimens (interferon versus DAAs) simultaneously with liver stiffness. Because early DAA roll-out prioritized advanced liver disease, treatment is structurally collinear with LSM. Consequently, regimen was excluded from the primary models to prevent multicollinearity and overfitting, prioritizing LSM as a continuous metric. However, sensitivity analyses substituting LSM for treatment regimen confirmed the stability of the primary immunological signatures. Similarly, while clinical obesity (BMI ≥ 30 kg/m^2^) is a known driver of systemic inflammation and immune senescence, its low prevalence in our cohort (15.6%) precluded its inclusion as a covariate in the multivariable models to prevent overfitting. Fifth, while sample availability restricted our analytical sub-cohorts, their baseline clinical profiles remained consistent with the total cohort, minimizing the risk of selection bias. Sixth, the absence of a parallel control group (e.g., HCV-monoinfected individuals or HCV-naïve PWH) precludes attributing the observed immune profiles solely to the HIV/HCV interaction. Finally, restricting the cohort to patients with advanced fibrosis limits the generalizability of these associations to individuals with mild liver disease. Additionally, the epidemiological predominance of HCV genotype 1 precluded adequately powered sub-analyses of genotype-specific immunological signatures. Furthermore, although the composite score captures overall cross-neutralizing breadth, we observed heterogeneity across specific assay genotypes (e.g., divergent Gt3a neutralizing capacity; Supplementary Table S3), consistent with its distinct neutralization profile.

## 4. Material and Methods

### 4.1. Study Design and Participants

We conducted a multicenter cross-sectional study involving 64 HIV/HCV-coinfected individuals selected from a parent retrospective cohort previously characterized for long-term HCV-specific antibody decline [6] (see Appendix A). Participants were evaluated five years after achieving sustained virologic response (SVR; defined as undetectable HCV-RNA 12 or 24 weeks post-treatment) following either interferon-based (2012–2013) or direct-acting antiviral (DAA) therapy (2015–2016). Inclusion criteria prior to HCV therapy required stable combination antiretroviral therapy (cART), suppressed HIV-RNA (<50 copies/mL) for ≥6 months, and a CD4^+^ T-cell count ≥200 cells/μL. Exclusion criteria included hepatitis B virus coinfection, acute HCV infection, documented HCV reinfection, and immunosuppressive therapy. Due to sample availability constraints (logistical delays preventing fresh blood processing within the required window for flow cytometry, or insufficient cryopreserved plasma volume for multiplex assays), the final analytical subgroups comprised 58 participants for cellular immunophenotyping and 50 for plasma biomarker quantification. This missingness occurred completely at random (MCAR) and was independent of clinical characteristics.

Epidemiological and clinical data were retrieved from the cohort databases [60,61]. The study was approved by the Ethics Committee of the Instituto de Salud Carlos III (CEI PI 23_2021), conducted in accordance with the Declaration of Helsinki, and followed STROBE guidelines (Supplementary Data S1). All participants provided written informed consent prior to enrollment.

### 4.2. Flow Cytometry and Immunophenotyping

We characterized T-cell subsets in fresh, K_2_-EDTA-anticoagulated whole blood using a validated 9-color antibody panel (Supplementary Table S1) on a Gallios™ flow cytometer (Beckman Coulter, Brea, CA, USA). As detailed in Supplementary Table S2, T-cell lineages (CD4^+^ and CD8^+^) were stratified into four differentiation stages based on CD45RA and CD28 expression [62]: Naïve, Central Memory (CM), Effector Memory (EM), and Terminally Differentiated Effector Memory re-expressing CD45RA (TemRA). Functional states were assessed by quantifying markers of activation (CD38, HLA-DR), associated with senescence (CD57), and survival/homeostatic potential (CD127). Gating strategies, including doublet exclusion and morphological quality control, are provided in Supplementary Data S2.

### 4.3. Enzyme-Linked Immunosorbent Multiplex Assays

We quantified 19 plasma biomarkers using a custom ProcartaPlex™ multiplex immunoassay (Bender MedSystems, Vienna, Austria) on a Luminex 200™ platform (Luminex Corporation, Austin, TX, USA), according to the manufacturer’s protocols. Analytes were stratified into three functional axes: (i) Co-stimulatory, comprising soluble cluster of differentiation (sCD27, sCD28, sCD80, OX40 (CD134), CD48 (BLAST-1)), inducible T-cell costimulator ligand (ICOS-L/B7-H2), B7 homolog 6 (B7-H6), and soluble glucocorticoid-induced TNF receptor-related protein (sGITR); (ii) Inhibitory, comprising soluble programmed death-1 (sPD-1), programmed death-ligand 1 (PD-L1), PD-L2, B- and T-lymphocyte attenuator (BTLA), V-domain Ig suppressor of T-cell activation (VISTA/B7-H5), T-cell immunoglobulin and mucin-domain containing-3 (TIM-3), soluble lymphocyte activation gene-3 (sLAG-3), and soluble cytotoxic T-lymphocyte-associated protein 4 (sCTLA-4); and (iii) Inflammatory, comprising soluble tumor necrosis factor receptor I (sTNF-RI), interleukin (IL)-8, and IL-18.

To maximize statistical power and avoid left-censoring bias, raw mean fluorescence intensity (MFI) was used as the primary continuous variable [63,64]. This approach preserves the continuous variance of low-end signals. While MFI provides a valid metric for internal cohort comparisons, these values cannot be directly compared across different external studies. To eliminate inter-assay variability, all samples were processed simultaneously in a single batch using the same reagent lot. Methodological details are provided in Supplementary Data S3.

### 4.4. Quantification of Anti-HCV-E2 Antibodies and HCV Neutralization

We quantified plasma antibody binding (HCV-E2Abs) and neutralizing capacity (HCV-nAbs) against a representative panel of five HCV genotypes (Gt1a, Gt1b, Gt2a, Gt3a, and Gt4a), as previously described [6,7]. Binding magnitude was assessed via indirect ELISA using recombinant E2 ectodomains. Neutralization potency was determined using cell-culture-derived HCV (HCVcc) chimeras expressing genotype-specific Core-NS2 sequences in microneutralization assays (see Supplementary Data S4). To derive continuous variables for statistical modeling, titration curves were fitted to calculate the Area Under the Curve (AUC) for each genotype. A composite score was generated for each participant by calculating the arithmetic mean of the five genotype-specific AUC values, providing a global metric for binding magnitude and neutralizing capacity. Pairwise correlations demonstrated strong internal consistency across most genotypes, alongside expected biological heterogeneity for Gt3a (Supplementary Table S3), supporting the composite score as a robust metric of cross-neutralizing breadth.

### 4.5. Statistical Analysis

Associations between immunological parameters and antibody composite scores (HCV-E2Abs and HCV-nAbs) were assessed using Generalized Linear Models (GLMs) with a gamma distribution and log-link function. This approach accounts for the positive skewness of antibody titer data without requiring prior transformation of the dependent variable. All continuous independent variables (T-cell subset frequencies and plasma biomarker MFI values) were log2-transformed prior to generalized linear modeling. Consequently, effect sizes are reported as adjusted Arithmetic Mean Ratios (aAMRs) with 95% Confidence Intervals (95% CIs), representing the multiplicative change in the dependent variable (antibody composite score) per two-fold increase in the independent variable.

All models were adjusted for a limited, predefined set of five clinical confounders to avoid overfitting: age, sex, liver stiffness measurement (LSM), HCV genotype (1 vs. non-1), and nadir CD4^+^ T-cell count. Age, LSM, and nadir CD4^+^ count were modeled as continuous variables to preserve data variance. Given the exploratory nature of this post hoc analysis and its goal of generating hypotheses, we applied a Benjamini–Hochberg False Discovery Rate (FDR) threshold of q < 0.10. All analyses were performed using Stata 17.0 (StataCorp, College Station, TX, USA).

## 5. Conclusions

In conclusion, our findings underscore that post-SVR immune normalization does not equate to functional humoral protection. Focal CD4^+^ T-cell activation is associated with higher titers of binding antibodies, whereas a residual “immunological scar,” characterized by widespread memory T-cell activation and the shedding of inhibitory ICPs, is associated with higher overall neutralizing capacity. Furthermore, inflammasome-driven innate inflammation (IL-18) and the restoration of the naïve CD8^+^ compartment are inversely associated with this neutralizing capacity. Collectively, these data indicate that long-term humoral immunity is embedded within a coordinated adaptive immune landscape rather than dependent on B-cell memory in isolation.

## Figures and Tables

**Table 1:** Characteristics of individuals with HIV/HCV coinfection at the beginning of HCV antiviral therapy.

Characteristics	Values (N = 64)
**Sex (male)**	51 (80%)
**Age (years)**	51 (48–54)
**BMI (kg/m^2^)**	24.7 (21.4–28.7)
BMI ≥ 25 kg/m^2^	31 (48%)
**Substance use**	
** Smoking status**	
Never	4 (6%)
Former (≥6 months)	18 (28%)
Current	42 (66%)
** High alcohol intake (>50 g/day)**	27 (42%)
** Intravenous drug use**	
Never	15 (23%)
Former (≥6 months)	49 (77%)
**HIV-related data**	
** Prior AIDS**	2 (3%)
** Nadir CD4^+^ T-cells (cells/mm^3^)**	130 (84–214)
Nadir CD4^+^ < 200 cells/mm^3^	47 (73%)
**Baseline CD4^+^ T-cells (cells/mm^3^)**	486 (282.5–698.0)
Baseline CD4^+^ < 500 cells/mm^3^	33 (52%)
**Antiretroviral therapy**	
NRTI + NNRTI-based	44 (76%)
NRTI + PI-based	13 (22%)
Other	1 (2%)
**HCV-related data**	
**Previous HCV therapy (IFNα + ribavirin)**	34 (53%)
**HCV genotype**	
1	47 (73%)
3	9 (14%)
4	7 (11%)
Other/Mixed	1 (2%)
**HCV RNA (Log_10_ IU/mL)**	6.1 (5.7–6.6)
HCV-RNA ≥ 850,000 IU/mL	39 (61%)
**Liver disease**	
**LSM (kPa)**	21.2 (12.9–34.6)
<12.5 kPa	14 (22%)
12.5–24.9 kPa	21 (33%)
25–39.9 kPa	19 (30%)
≥40 kPa	10 (15%)

**Statistics**: Data are presented as n (%) for categorical variables and median (interquartile range, IQR) for continuous variables. **Abbreviations**: HIV, human immunodeficiency virus; HCV, hepatitis C virus; BMI, body mass index; AIDS, acquired immune deficiency syndrome; NRTI, nucleoside reverse transcriptase inhibitor; NNRTI, non-nucleoside reverse transcriptase inhibitor; PI, protease inhibitor; IFNα, interferon-alpha; LSM, liver stiffness measurement.

**Table 2:** Adjusted associations between CD4^+^ T-cell subsets and anti-E2 (HCV-E2Abs) and neutralizing (HCV-nAbs) antibody titers five years post-SVR.

		HCV-E2Abs			HCV-nAbs		
T-Cell Subset	Markers	aAMR (95% CI)	*p*-Value	q-Value	aAMR (95% CI)	*p*-Value	q-Value
**Total—Base Population**	**CD38^+^**	**1.58 (1.19; 2.10)**	**0.002**	**0.028**	**2.05 (1.14; 3.69)**	**0.017**	**0.079**
*(CD4^+^)*	HLA-DR^+^	1.20 (0.97; 1.48)	0.091	0.220	1.51 (0.82; 2.79)	0.184	0.321
	**CD38^+^ HLA-DR^+^**	**1.24 (1.06; 1.46)**	**0.008**	**0.037**	1.32 (0.85; 2.06)	0.216	0.321
	**CD57^+^**	1.18 (0.98; 1.41)	0.087	0.220	**1.54 (1.06; 2.24)**	**0.022**	**0.087**
	CD127^+^	1.56 (0.67; 3.60)	0.300	0.525	1.86 (0.85; 4.08)	0.119	0.222
**Naïve CD4^+^**	Naïve	0.98 (0.77; 1.24)	0.847	0.893	0.58 (0.33; 1.01)	0.054	0.126
*(CD4^+^CD45RA^+^CD28^+^)*	**CD38^+^**	**1.33 (1.15; 1.54)**	**<0.001**	**0.003**	**2.29 (1.61; 3.25)**	**<0.001**	**0.003**
	HLA-DR^+^	1.08 (0.94; 1.23)	0.293	0.525	1.38 (0.97; 1.95)	0.070	0.151
	**CD38^+^ HLA-DR^+^**	**1.13 (1.04; 1.24)**	**0.006**	**0.034**	**1.42 (1.08; 1.87)**	**0.012**	**0.073**
	**CD127^+^**	**2.74 (1.10; 6.80)**	**0.030**	0.105	1.55 (0.43; 5.56)	0.503	0.571
**Central Memory (CM)**	CM	1.04 (0.69; 1.56)	0.861	0.893	0.67 (0.20; 2.20)	0.510	0.571
*(CD4^+^CD45RA-CD28^+^)*	**CD38^+^**	**1.31 (1.09; 1.58)**	**0.004**	**0.034**	**2.14 (1.22; 3.75)**	**0.008**	**0.073**
	**HLA-DR^+^**	1.08 (0.89; 1.31)	0.414	0.682	**1.70 (1.03; 2.81)**	**0.039**	**0.099**
	**CD38^+^ HLA-DR^+^**	1.14 (0.98; 1.34)	0.099	0.220	**1.79 (1.13; 2.82)**	**0.013**	**0.073**
	CD57^+^	1.03 (0.83; 1.29)	0.772	0.874	1.44 (0.82; 2.53)	0.205	0.321
	CD127^+^	1.18 (0.37; 3.71)	0.780	0.874	3.19 (0.37; 27.53)	0.292	0.389
**Effector Memory (EM)**	EM	1.05 (0.88; 1.26)	0.568	0.757	1.22 (0.77; 1.93)	0.402	0.489
*(CD4^+^CD45RA-CD28^−^)*	CD38^+^	1.15 (0.96; 1.37)	0.132	0.264	1.09 (0.76; 1.58)	0.637	0.686
	**HLA-DR^+^**	1.04 (0.86; 1.26)	0.670	0.816	**0.59 (0.36; 0.96)**	**0.032**	**0.090**
	CD38^+^ HLA-DR^+^	1.06 (0.91; 1.22)	0.460	0.716	0.78 (0.52; 1.16)	0.218	0.321
	CD57^+^	1.16 (0.98; 1.37)	0.087	0.220	1.18 (0.83; 1.68)	0.365	0.465
	CD127^+^	0.94 (0.73; 1.21)	0.634	0.807	1.09 (0.68; 1.74)	0.717	0.744
**TemRA**	TemRA	0.99 (0.81; 1.21)	0.941	0.941	0.96 (0.67; 1.39)	0.844	0.844
*(CD4^+^CD45RA^+^CD28^−^)*	**CD38** ^+^	**1.24 (1.07; 1.44)**	**0.005**	**0.034**	**1.55 (1.05; 2.30)**	**0.028**	**0.087**
	HLA-DR^+^	1.12 (0.98; 1.29)	0.102	0.220	1.34 (0.79; 2.25)	0.275	0.385
	**CD38^+^ HLA-DR^+^**	**1.15 (1.03; 1.28)**	**0.013**	**0.052**	1.43 (0.95; 2.15)	0.087	0.174
	**CD57^+^**	1.05 (0.90; 1.23)	0.520	0.736	**1.49 (1.05; 2.10)**	**0.026**	**0.087**
	**CD127^+^**	1.06 (0.88; 1.28)	0.526	0.736	**1.58 (1.22; 2.05)**	**0.001**	**0.014**

**Statistics**: Data are presented as adjusted Arithmetic Mean Ratios (aAMRs) and 95% Confidence Intervals (95% CIs). Values were derived from Generalized Linear Models (GLMs) with a gamma distribution and log-link function, for a fixed set of clinical confounders (age, sex, liver stiffness, HCV genotype, and nadir CD4^+^ T-cell count). aAMR represents the multiplicative effect on antibody titers per doubling (log2-increase) of the T-cell subset frequency. Values in bold indicate statistically significant associations (FDR-corrected q-value < 0.10). **Abbreviations**: aAMR, adjusted Arithmetic Mean Ratio; CM, Central Memory; EM, Effector Memory; FDR, False Discovery Rate; HCV, Hepatitis C Virus; nAbs, neutralizing antibodies; SVR, Sustained Virologic Response; TemRA, Terminally Differentiated Effector Memory re-expressing CD45RA.

**Table 3:** Adjusted associations between CD8^+^ T-cell subsets and anti-E2 (HCV-E2Abs) and neutralizing (HCV-nAbs) antibody titers five years post-SVR.

		HCV-E2Abs			HCV-nAbs		
T-Cell Subset	Markers	aAMR (95% CI)	*p*-Value	q-Value	aAMR (95% CI)	*p*-Value	q-Value
**Total—Base Population**	CD38^+^	1.18 (1.04; 1.33)	0.008	0.168	1.40 (0.96; 2.05)	0.083	0.178
*(CD8^+^)*	HLA-DR^+^	1.13 (0.97; 1.31)	0.118	0.395	1.59 (1.02; 2.50)	0.042	0.118
	CD38^+^ HLA-DR^+^	1.14 (1.01; 1.29)	0.040	0.224	1.42 (0.93; 2.18)	0.109	0.196
	CD57^+^	1.21 (0.94; 1.55)	0.141	0.395	1.78 (0.92; 3.47)	0.089	0.178
	CD127^+^	0.83 (0.47; 1.45)	0.506	0.604	0.60 (0.20; 1.82)	0.364	0.510
**Naïve CD8^+^**	**Naïve**	0.86 (0.72; 1.04)	0.119	0.395	**0.41 (0.23; 0.73)**	**0.003**	**0.042**
*(CD8^+^CD45RA^+^CD28^+^)*	**CD38^+^**	1.10 (1.02; 1.19)	0.018	0.168	**1.17 (1.04; 1.32)**	**0.012**	**0.056**
	HLA-DR^+^	1.07 (0.95; 1.21)	0.294	0.488	1.10 (0.80; 1.52)	0.550	0.620
	CD38^+^ HLA-DR^+^	1.08 (1.02; 1.14)	0.013	0.168	1.17 (1.00; 1.38)	0.051	0.130
	CD127^+^	1.33 (0.70; 2.56)	0.385	0.570	2.64 (0.73; 9.48)	0.137	0.226
**Central Memory (CM)**	CM	0.90 (0.70; 1.15)	0.387	0.570	0.79 (0.40; 1.56)	0.500	0.609
*(CD8^+^CD45RA-CD28^+^)*	CD38^+^	1.03 (0.87; 1.20)	0.756	0.784	1.13 (0.76; 1.68)	0.554	0.620
	**HLA-DR^+^**	1.13 (0.94; 1.36)	0.204	0.408	**2.17 (1.26; 3.76)**	**0.005**	**0.045**
	CD38^+^ HLA-DR^+^	1.05 (0.91; 1.21)	0.531	0.604	1.26 (0.86; 1.85)	0.229	0.337
	CD57^+^	1.09 (0.92; 1.30)	0.295	0.488	1.68 (0.83; 3.41)	0.153	0.238
	CD127^+^	1.36 (0.61; 3.02)	0.457	0.604	2.08 (0.32; 13.36)	0.440	0.565
**Effector Memory (EM)**	**EM**	1.14 (0.93; 1.39)	0.201	0.408	**1.57 (1.08; 2.28)**	**0.017**	**0.060**
*(CD8^+^CD45RA-CD28^−^)*	CD38^+^	1.09 (0.98; 1.22)	0.121	0.395	1.47 (0.98; 2.19)	0.064	0.149
	**HLA-DR^+^**	1.09 (0.93; 1.26)	0.296	0.488	**1.80 (1.17; 2.76)**	**0.007**	**0.045**
	**CD38^+^ HLA-DR^+^**	1.09 (0.97; 1.22)	0.170	0.408	**1.53 (1.08; 2.15)**	**0.016**	**0.060**
	CD57^+^	1.27 (0.93; 1.74)	0.134	0.395	2.11 (0.84; 5.32)	0.112	0.196
	CD127^+^	0.92 (0.70; 1.20)	0.545	0.604	0.97 (0.54; 1.73)	0.920	0.951
**TemRA**	TemRA	1.10 (0.85; 1.44)	0.466	0.604	1.08 (0.57; 2.04)	0.824	0.887
*(CD8^+^CD45RA^+^CD28^−^)*	**CD38^+^**	1.10 (1.01; 1.20)	0.035	0.224	**1.47 (1.19; 1.81)**	**<0.001**	**0.003**
	**HLA-DR^+^**	1.00 (0.94; 1.05)	0.879	0.879	**1.16 (1.02; 1.32)**	**0.025**	**0.078**
	**CD38^+^ HLA-DR^+^**	1.02 (0.95; 1.09)	0.561	0.604	**1.21 (1.05; 1.40)**	**0.008**	**0.045**
	CD57^+^	1.22 (0.91; 1.65)	0.186	0.408	1.47 (0.55; 3.93)	0.444	0.565
	CD127^+^	0.93 (0.76; 1.16)	0.536	0.604	0.98 (0.58; 1.67)	0.951	0.951

**Statistics**: Data are presented as adjusted Arithmetic Mean Ratios (aAMRs) and 95% Confidence Intervals (95% CIs). Values were derived from Generalized Linear Models (GLMs) with a gamma distribution and log-link function, for a fixed set of clinical confounders (age, sex, liver stiffness, HCV genotype, and nadir CD4^+^ T-cell count). aAMR represents the multiplicative effect on antibody titers per doubling (log2-increase) of the T-cell subset frequency. Values in bold indicate statistically significant associations (FDR-corrected q-value < 0.10). **Abbreviations**: aAMR, adjusted Arithmetic Mean Ratio; CM, Central Memory; EM, Effector Memory; FDR, False Discovery Rate; HCV, Hepatitis C Virus; nAbs, neutralizing antibodies; SVR, Sustained Virologic Response; TemRA, Terminally Differentiated Effector Memory re-expressing CD45RA.

**Table 4:** Adjusted associations between plasma biomarkers and anti-E2 (HCV-E2Abs) and neutralizing (HCV-nAbs) antibody titers five years post-SVR.

	HCV-E2Abs			HCV-nAbs		
Biomarker (MFI)	aAMR (95% CI)	*p*-Value	q-Value	aAMR (95% CI)	*p*-Value	q-Value
** A. Co-stimulatory Axis**						
** sCD27**	1.46 (1.15; 1.85)	** 0.002**	** 0.038**	2.11 (0.84; 5.32)	0.112	0.213
** sCD28**	0.97 (0.81; 1.15)	0.724	0.815	1.68 (1.19; 2.38)	** 0.003**	** 0.029**
sCD80	0.95 (0.79; 1.14)	0.604	0.815	1.48 (1.01; 2.17)	0.045	0.143
sOX40 (CD134)	1.29 (0.92; 1.80)	0.137	0.325	2.25 (0.71; 7.14)	0.169	0.269
sCD48 (BLAST-1)	0.97 (0.76; 1.24)	0.835	0.835	0.98 (0.56; 1.70)	0.930	0.930
sICOS-L (B7-H2)	1.27 (0.83; 1.95)	0.272	0.517	1.88 (0.80; 4.43)	0.147	0.269
sB7-H6	1.10 (0.71; 1.70)	0.658	0.815	0.54 (0.25; 1.14)	0.105	0.213
sGITR	0.96 (0.77; 1.18)	0.674	0.815	1.55 (1.01; 2.38)	0.047	0.149
** B. Inhibitory Axis**						
sPD-1	1.02 (0.85; 1.23)	0.819	0.835	1.46 (0.94; 2.27)	0.092	0.213
sPD-L1	0.94 (0.84; 1.06)	0.311	0.581	1.07 (0.40; 2.84)	0.899	0.930
** sPD-L2**	1.12 (0.80; 1.55)	0.518	0.815	2.50 (1.29; 4.82)	** 0.006**	** 0.048**
sBTLA	0.97 (0.83; 1.14)	0.728	0.815	1.29 (0.81; 2.07)	0.284	0.358
sVISTA (B7-H5)	1.00 (0.85; 1.18)	0.992	0.992	1.17 (0.67; 2.04)	0.571	0.601
sTIM-3	1.19 (0.90; 1.57)	0.225	0.428	1.98 (1.00; 3.89)	0.049	0.155
** sLAG-3**	1.06 (0.82; 1.37)	0.665	0.815	1.97 (1.17; 3.30)	** 0.010**	** 0.063**
** sCTLA-4**	0.99 (0.85; 1.17)	0.932	0.992	1.46 (1.07; 1.99)	** 0.018**	** 0.086**
** C. Inflammatory Context**						
** sTNF-RI**	1.12 (0.97; 1.29)	0.109	0.325	1.44 (1.16; 1.79)	** 0.001**	** 0.019**
IL-8	1.17 (0.94; 1.45)	0.163	0.355	1.16 (0.66; 2.03)	0.616	0.648
** IL-18**	0.98 (0.85; 1.14)	0.829	0.835	0.53 (0.36; 0.78)	** 0.001**	** 0.019**

**Statistics**: Data are presented as adjusted Arithmetic Mean Ratios (aAMRs) and 95% Confidence Intervals (95% CIs). Values were derived from Generalized Linear Models (GLMs) with a gamma distribution and log-link function, for a fixed set of clinical confounders (age, sex, liver stiffness, HCV genotype, and nadir CD4^+^ T-cell count). aAMR represents the multiplicative effect on antibody titers per doubling (log2-increase) of the biomarker level. Values in bold indicate statistically significant associations (FDR-corrected q-value < 0.10). **Abbreviations:** aAMR, adjusted Arithmetic Mean Ratio; sB7-H6, soluble Natural cytotoxicity triggering receptor 3 ligand 1; sBTLA, soluble B- and T-lymphocyte attenuator; FDR, False Discovery Rate; HCV, Hepatitis C Virus; sICOS-L, soluble Inducible T-cell costimulator ligand; IL, Interleukin; nAbs, neutralizing antibodies; sOX40 (CD134), soluble Tumor necrosis factor receptor superfamily member 4; sPD-L1, soluble Programmed death-ligand 1; sPD-L2, soluble Programmed death-ligand 2; sCD27, Soluble Cluster of Differentiation 27; sTNF-RI, Soluble Tumor Necrosis Factor Receptor I; SVR, Sustained Virological Response; sTIM-3, soluble T-cell immunoglobulin and mucin-domain containing-3; sVISTA, soluble V-domain Ig suppressor of T cell activation; MFI, mean fluorescence intensity.

## Data Availability

The original contributions presented in this study are included in the article/Supplementary Material. Further inquiries can be directed to the corresponding authors.

## Supplementary Materials

The following supporting information can be downloaded at: https://www.mdpi.com/article/10.3390/ph19060854/s1, Data S1: STROBE Statement—checklist of items that should be included in reports of observational studies; Data S2: Flow Cytometry and Gating Strategy; Data S3: Plasma Biomarker Quantification (Luminex Technology); Data S4: Quantification of Humoral Immune Responses; Data S5: List of Abbreviations; Table S1: List of monoclonal antibodies used for flow cytometry immunophenotyping; Table S2: Phenotypic definitions and functional markers of analyzed T-cell subsets; Table S3: Pairwise correlation analysis of antibody responses across the five tested HCV genotypes and their composite score; Table S4: Clinical characteristics of the total cohort and analytical sub-groups; Table S5: Unadjusted associations between CD4^+^ T-cell subsets and HCV-specific antibody responses five years post-SVR (n = 58); Table S6: Sensitivity analysis of CD4^+^ T-cell subsets associated HCV-E2Abs and HCV-nAbs titers five years post-SVR substituting LSM with HCV treatment regimen; Table S7: Unadjusted associations between CD8^+^ T-cell subsets and HCV-specific antibody responses five years post-SVR (n = 58); Table S8: Sensitivity analysis of CD8^+^ T-cell subsets associated HCV-E2Abs and HCV-nAbs titers five years post-SVR substituting LSM with HCV treatment regimen; Table S9: Unadjusted associations of plasma biomarkers with HCV-E2Abs and HCV-nAbs titers five years post-SVR (n = 50); Table S10: Sensitivity analysis of plasma biomarkers associated HCV-E2Abs and HCV-nAbs titers five years post-SVR substituting LSM with HCV treatment regimen. Refs. [7,56,63,64,65,66,67,68,69,70,71] cited in Supplementary Materials.

## Appendix A

**The GESIDA 3603b Cohort Study Group**

***Hospital General Universitario Gregorio Marañón, Madrid:*** A Carrero, P Miralles, JC López, F Parras, B Padilla, T Aldamiz-Echevarría, F Tejerina, C Díez, L Pérez-Latorre, C Fanciulli, I Gutiérrez, M Ramírez, S Carretero, JM Bellón, J Bermejo, and J Berenguer.

***Hospital Universitario La Paz, Madrid:*** V Hontañón, JR Arribas, ML Montes, I Bernardino, JF Pascual, F Zamora, JM Peña, F Arnalich, M Díaz, J González-García.

***Hospital de la Santa Creu i Sant Pau, Barcelona:*** P Domingo, JM Guardiola.

***Hospital Universitari Vall d’Hebron, Barcelona:*** E Van den Eynde, M Pérez, E Ribera, M Crespo.

***Hospital Universitario Ramón y Cajal, Madrid:*** JL Casado, F Dronda, A Moreno, MJ Pérez-Elías, MA Sanfrutos, S Moreno, C Quereda.

***Hospital Universitario Príncipe de Asturias, Alcalá de Henares:*** A Arranz, E Casas, J de Miguel, S Schroeder, J Sanz.

***Hospital Universitario de La Princesa, Madrid:*** J Sanz, I Santos.

***Hospital Donostia, San Sebastián:*** MJ Bustinduy, JA Iribarren, F Rodríguez-Arrondo, MA Von-Wichmann.

***Hospital Clínico San Carlos, Madrid:*** J Vergas, MJ Téllez.

**Hospital Universitario San Cecilio, Granada**: D. Vinuesa, L. Muñoz, and J. Hernández-Quero.

***Hospital Clínico Universitario, Valencia:*** A Ferrer, MJ Galindo.

***Hospital General Universitario, Valencia:*** L Ortiz, E Ortega.

***Hospital Universitari La Fe, Valencia:*** M Montero, M Blanes, S Cuellar, J Lacruz, M Salavert, J López-Aldeguer.

***Hospital Universitario de Getafe, Getafe:*** G Pérez, G Gaspar.

***Fundación SEIMC-GESIDA, Madrid:*** M Yllescas, P Crespo, E Aznar, H Esteban.

**The ESCORIAL study group**

***Hospital General Universitario Gregorio Marañón*** (Madrid, Spain): Cristina Díez, Luis Ibáñez, Leire Pérez-Latorre, Diego Rincón, Teresa Aldámiz-Echevarría, Vega Catalina, Pilar Miralles, Teresa Aldámiz-Echevarría, Francisco Tejerina, María C Gómez-Rico, Esther Alonso, José M Bellón, Rafael Bañares, and Juan Berenguer.

***Hospital Universitario La Paz/IdiPAZ*** (Madrid, Spain): José Arribas, José I Bernardino, Ana Delgado, Carmen Busca, Javier García-Samaniego, Víctor Hontañón, Luz Martín-Carbonero, Rafael Micán, María L Montes-Ramírez, Victoria Moreno, Antonio Olveira, Ignacio Pérez-Valero, Eulalia valencia, and Juan González-García.

***Hospital Universitario Puerta de Hierro*** (Madrid, Spain): Elba Llop and José Luis Calleja.

***Hospital Universitario Ramón y Cajal*** (Madrid, Spain): Javier Martínez and Agustín Albillos.

***Fundación SEIMC/GeSIDA*** (Madrid, Spain): Marta de Miguel, María Yllescas, and Herminia Esteban.

## References

[1] Pawlotsky J.M., Negro F., Aghemo A., Berenguer M., Dalgard O., Dusheiko G., Marra F., Puoti M., Wedemeyer H., European Association for the Study of the Liver (2020). EASL recommendations on treatment of hepatitis C: Final update of the series. J. Hepatol..

[2] Martinello M., Solomon S.S., Terrault N.A., Dore G.J. (2023). Hepatitis C. Lancet.

[3] Brochado O., Martinez I., Berenguer J., Medrano L., Gonzalez-Garcia J., Jimenez-Sousa M.A., Carrero A., Hontanon V., Navarro J., Guardiola J.M. (2021). HCV eradication with IFN-based therapy does not completely restore gene expression in PBMCs from HIV/HCV-coinfected patients. J. Biomed. Sci..

[4] Albillos A., Martin-Mateos R., Van der Merwe S., Wiest R., Jalan R., Alvarez-Mon M. (2022). Cirrhosis-associated immune dysfunction. Nat. Rev. Gastroenterol. Hepatol..

[5] Osuch S., Metzner K.J., Caraballo Cortes K. (2020). Reversal of T Cell Exhaustion in Chronic HCV Infection. Viruses.

[6] Sepulveda-Crespo D., Volpi C., Amigot-Sanchez R., Yelamos M.B., Diez C., Gomez J., Hontanon V., Berenguer J., Gonzalez-Garcia J., Martin-Escolano R. (2024). Sustained Long-Term Decline in Anti-HCV Neutralizing Antibodies in HIV/HCV-Coinfected Patients Five Years after HCV Therapy: A Retrospective Study. Pharmaceuticals.

[7] Sepulveda-Crespo D., Yelamos M.B., Diez C., Gomez J., Hontanon V., Torresano-Felipe F., Berenguer J., Gonzalez-Garcia J., Ibanez-Samaniego L., Llop E. (2022). Negative impact of HIV infection on broad-spectrum anti-HCV neutralizing antibody titers in HCV-infected patients with advanced HCV-related cirrhosis. Biomed. Pharmacother..

[8] Bailey J.R., Dowd K.A., Snider A.E., Osburn W.O., Mehta S.H., Kirk G.D., Thomas D.L., Ray S.C. (2015). CD4^+^ T-Cell-Dependent Reduction in Hepatitis C Virus-Specific Neutralizing Antibody Responses After Coinfection with Human Immunodeficiency Virus. J. Infect. Dis..

[9] Deeks S.G., Tracy R., Douek D.C. (2013). Systemic effects of inflammation on health during chronic HIV infection. Immunity.

[10] Khodadadi L., Cheng Q., Radbruch A., Hiepe F. (2019). The Maintenance of Memory Plasma Cells. Front. Immunol..

[11] Wang P., Su N., Yan X., Xu F., Zhang Y. (2025). Circulating plasma cells: From basic mechanisms to clinical applications. Front. Immunol..

[12] Leno-Duran E., Serrano-Conde E., Salas-Rodriguez A., Salcedo-Bellido I., Barrios-Rodriguez R., Fuentes A., Vinuela L., Garcia F., Requena P. (2024). Evaluation of inflammatory biomarkers and their association with anti-SARS-CoV-2 antibody titers in healthcare workers vaccinated with BNT162B2. Front. Immunol..

[13] Shah H.B., Joshi S.K., Rampuria P., Devera T.S., Lang G.A., Stohl W., Lang M.L. (2013). BAFF- and APRIL-dependent maintenance of antibody titers after immunization with T-dependent antigen and CD1d-binding ligand. J. Immunol..

[14] Jourdan M., Cren M., Robert N., Bollore K., Fest T., Duperray C., Guilloton F., Hose D., Tarte K., Klein B. (2014). IL-6 supports the generation of human long-lived plasma cells in combination with either APRIL or stromal cell-soluble factors. Leukemia.

[15] Paludan S.R., Pradeu T., Masters S.L., Mogensen T.H. (2021). Constitutive immune mechanisms: Mediators of host defence and immune regulation. Nat. Rev. Immunol..

[16] Lightman S.M., Utley A., Lee K.P. (2019). Survival of Long-Lived Plasma Cells (LLPC): Piecing Together the Puzzle. Front. Immunol..

[17] Aaron T., Laudermilch E., Benet Z., Ovando L.J., Chandran K., Fooksman D. (2023). TNF-alpha Limits Serological Memory by Disrupting the Bone Marrow Niche. J. Immunol..

[18] De Milito A., Aleman S., Marenzi R., Sonnerborg A., Fuchs D., Zazzi M., Chiodi F. (2002). Plasma levels of soluble CD27: A simple marker to monitor immune activation during potent antiretroviral therapy in HIV-1-infected subjects. Clin. Exp. Immunol..

[19] Schafer A.L., Ruiz-Aparicio P.F., Kraemer A.N., Chevalier N. (2023). Crosstalk in the diseased plasma cell niche-the force of inflammation. Front. Immunol..

[20] Park S.B., Zimmer-Harwood P., Liang T.J. (2026). Targets of protective immunity and opportunities in hepatitis C virus vaccine development. Nat. Rev. Immunol..

[21] Grimsholm O. (2023). CD27 on human memory B cells-more than just a surface marker. Clin. Exp. Immunol..

[22] Traggiai E., Puzone R., Lanzavecchia A. (2003). Antigen dependent and independent mechanisms that sustain serum antibody levels. Vaccine.

[23] Brenchley J.M., Karandikar N.J., Betts M.R., Ambrozak D.R., Hill B.J., Crotty L.E., Casazza J.P., Kuruppu J., Migueles S.A., Connors M. (2003). Expression of CD57 defines replicative senescence and antigen-induced apoptotic death of CD8^+^ T cells. Blood.

[24] Tedeschi V., Paldino G., Kunkl M., Paroli M., Sorrentino R., Tuosto L., Fiorillo M.T. (2022). CD8^+^ T Cell Senescence: Lights and Shadows in Viral Infections, Autoimmune Disorders and Cancer. Int. J. Mol. Sci..

[25] Autaa G., Korenkov D., van Beek J., Pellegrin I., Parfait B., van Baarle D., Launay O., Tartour E., Appay V. (2025). Re-evaluating CD57 as a marker of T cell senescence: Implications for immune ageing and differentiation. Immun. Ageing.

[26] Le Priol Y., Puthier D., Lecureuil C., Combadiere C., Debre P., Nguyen C., Combadiere B. (2006). High cytotoxic and specific migratory potencies of senescent CD8^+^ CD57^+^ cells in HIV-infected and uninfected individuals. J. Immunol..

[27] Palmer C.D., Romero-Tejeda M., Scully E.P., Lockhart A., Seaman M.S., Goldenthal A., Piechocka-Trocha A., Walker B.D., Chibnik L.B., Jost S. (2016). Increased frequencies of CD8^+^CD57^+^ T cells are associated with antibody neutralization breadth against HIV in viraemic controllers. J. Int. AIDS Soc..

[28] Zicari S., Sessa L., Cotugno N., Ruggiero A., Morrocchi E., Concato C., Rocca S., Zangari P., Manno E.C., Palma P. (2019). Immune Activation, Inflammation, and Non-AIDS Co-Morbidities in HIV-Infected Patients under Long-Term ART. Viruses.

[29] Younas M., Psomas C., Reynes C., Cezar R., Kundura L., Portales P., Merle C., Atoui N., Fernandez C., Le Moing V. (2019). Microbial Translocation Is Linked to a Specific Immune Activation Profile in HIV-1-Infected Adults with Suppressed Viremia. Front. Immunol..

[30] Poirier N., Chevalier M., Mary C., Hervouet J., Minault D., Baker P., Ville S., Le Bas-Bernardet S., Dilek N., Belarif L. (2016). Selective CD28 Antagonist Blunts Memory Immune Responses and Promotes Long-Term Control of Skin Inflammation in Nonhuman Primates. J. Immunol..

[31] Morita R., Schmitt N., Bentebibel S.E., Ranganathan R., Bourdery L., Zurawski G., Foucat E., Dullaers M., Oh S., Sabzghabaei N. (2011). Human blood CXCR5^+^CD4^+^ T cells are counterparts of T follicular cells and contain specific subsets that differentially support antibody secretion. Immunity.

[32] Lv Y., Ricard L., Gaugler B., Huang H., Ye Y. (2022). Biology and clinical relevance of follicular cytotoxic T cells. Front. Immunol..

[33] Strittmatter-Keller U., Walter C., Rauld C., Egli N., Regairaz C., Rabe S., Zenke G., Carballido J., Schweighoffer T. (2018). Fingerprints of CD8^+^ T cells on human pre-plasma and memory B cells. PLoS ONE.

[34] Cubas R.A., Mudd J.C., Savoye A.L., Perreau M., van Grevenynghe J., Metcalf T., Connick E., Meditz A., Freeman G.J., Abesada-Terk G. (2013). Inadequate T follicular cell help impairs B cell immunity during HIV infection. Nat. Med..

[35] Tzarum N., Giang E., Kong L., He L., Prentoe J., Augestad E., Hua Y., Castillo S., Lauer G.M., Bukh J. (2019). Genetic and structural insights into broad neutralization of hepatitis C virus by human V(H)1-69 antibodies. Sci. Adv..

[36] Radbruch A., Muehlinghaus G., Luger E.O., Inamine A., Smith K.G., Dorner T., Hiepe F. (2006). Competence and competition: The challenge of becoming a long-lived plasma cell. Nat. Rev. Immunol..

[37] Lindquist R.L., Niesner R.A., Hauser A.E. (2019). In the Right Place, at the Right Time: Spatiotemporal Conditions Determining Plasma Cell Survival and Function. Front. Immunol..

[38] Simons B.D., Karin O. (2024). Tuning of plasma cell lifespan by competition explains the longevity and heterogeneity of antibody persistence. Immunity.

[39] Robinson M.J., Webster R.H., Tarlinton D.M. (2020). How intrinsic and extrinsic regulators of plasma cell survival might intersect for durable humoral immunity. Immunol. Rev..

[40] Chiu C.Y., Schou M.D., McMahon J.H., Deeks S.G., Fromentin R., Chomont N., Wykes M.N., Rasmussen T.A., Lewin S.R. (2023). Soluble immune checkpoints as correlates for HIV persistence and T cell function in people with HIV on antiretroviral therapy. Front. Immunol..

[41] Kong Y., Wang Y., Wu X., Han J., Li G., Hua M., Han K., Zhang H., Li A., Zeng H. (2020). Storm of soluble immune checkpoints associated with disease severity of COVID-19. Signal Transduct. Target. Ther..

[42] Martin-Escolano R., Virseda-Berdices A., Berenguer J., Gonzalez-Garcia J., Brochado-Kith O., Fernandez-Rodriguez A., Diez C., Hontanon V., Resino S., Jimenez-Sousa M.A. (2024). Immune checkpoint proteins are associated with persistently high liver stiffness after successful HCV treatment in people with HIV: A retrospective study. Front. Immunol..

[43] Abdel-Hakeem M.S., Manne S., Beltra J.C., Stelekati E., Chen Z., Nzingha K., Ali M.A., Johnson J.L., Giles J.R., Mathew D. (2021). Epigenetic scarring of exhausted T cells hinders memory differentiation upon eliminating chronic antigenic stimulation. Nat. Immunol..

[44] Hensel N., Gu Z., Sagar, Wieland D., Jechow K., Kemming J., Llewellyn-Lacey S., Gostick E., Sogukpinar O., Emmerich F. (2021). Memory-like HCV-specific CD8^+^ T cells retain a molecular scar after cure of chronic HCV infection. Nat. Immunol..

[45] Baldanzi G. (2022). Immune Checkpoint Receptors Signaling in T Cells. Int. J. Mol. Sci..

[46] Horn L.A., Long T.M., Atkinson R., Clements V., Ostrand-Rosenberg S. (2018). Soluble CD80 Protein Delays Tumor Growth and Promotes Tumor-Infiltrating Lymphocytes. Cancer Immunol. Res..

[47] Van Zee K.J., Kohno T., Fischer E., Rock C.S., Moldawer L.L., Lowry S.F. (1992). Tumor necrosis factor soluble receptors circulate during experimental and clinical inflammation and can protect against excessive tumor necrosis factor alpha in vitro and in vivo. Proc. Natl. Acad. Sci. USA.

[48] Fooksman D.R., Jing Z., Park R. (2024). New insights into the ontogeny, diversity, maturation and survival of long-lived plasma cells. Nat. Rev. Immunol..

[49] Shrivastava S., Mukherjee A., Ray R., Ray R.B. (2013). Hepatitis C virus induces interleukin-1beta (IL-1beta)/IL-18 in circulatory and resident liver macrophages. J. Virol..

[50] Knorr J., Kaufmann B., Inzaugarat M.E., Holtmann T.M., Geisler L., Hundertmark J., Kohlhepp M.S., Boosheri L.M., Chilin-Fuentes D.R., Birmingham A. (2023). Interleukin-18 signaling promotes activation of hepatic stellate cells in mouse liver fibrosis. Hepatology.

[51] Taru V., Szabo G., Mehal W., Reiberger T. (2024). Inflammasomes in chronic liver disease: Hepatic injury, fibrosis progression and systemic inflammation. J. Hepatol..

[52] Doitsh G., Galloway N.L., Geng X., Yang Z., Monroe K.M., Zepeda O., Hunt P.W., Hatano H., Sowinski S., Munoz-Arias I. (2014). Cell death by pyroptosis drives CD4 T-cell depletion in HIV-1 infection. Nature.

[53] Xia P., Xing X.D., Yang C.X., Liao X.J., Liu F.H., Huang H.H., Zhang C., Song J.W., Jiao Y.M., Shi M. (2022). Activation-induced pyroptosis contributes to the loss of MAIT cells in chronic HIV-1 infected patients. Mil. Med. Res..

[54] Medrano L.M., Garcia-Broncano P., Berenguer J., Gonzalez-Garcia J., Jimenez-Sousa M.A., Guardiola J.M., Crespo M., Quereda C., Sanz J., Canorea I. (2018). Elevated liver stiffness is linked to increased biomarkers of inflammation and immune activation in HIV/hepatitis C virus-coinfected patients. AIDS.

[55] Medrano L.M., Berenguer J., Salguero S., Gonzalez-Garcia J., Diez C., Hontanon V., Garcia-Broncano P., Ibanez-Samaniego L., Bellon J.M., Jimenez-Sousa M.A. (2021). Successful HCV Therapy Reduces Liver Disease Severity and Inflammation Biomarkers in HIV/HCV-Coinfected Patients with Advanced Cirrhosis: A Cohort Study. Front. Med..

[56] Garcia-Broncano P., Medrano L.M., Berenguer J., Brochado-Kith O., Gonzalez-Garcia J., Jimenez-Sousa M.A., Quereda C., Sanz J., Tellez M.J., Diaz L. (2020). Mild profile improvement of immune biomarkers in HIV/HCV-coinfected patients who removed hepatitis C after HCV treatment: A prospective study. J. Infect..

[57] Nishio A., Hasan S., Park H., Park N., Salas J.H., Salinas E., Kardava L., Juneau P., Frumento N., Massaccesi G. (2022). Serum neutralization activity declines but memory B cells persist after cure of chronic hepatitis C. Nat. Commun..

[58] Hosseini-Hooshyar S., Hajarizadeh B., Bajis S., Law M., Janjua N.Z., Fierer D.S., Chromy D., Rockstroh J.K., Martin T.C.S., Ingiliz P. (2022). Risk of hepatitis C reinfection following successful therapy among people living with HIV: A global systematic review, meta-analysis, and meta-regression. Lancet HIV.

[59] Yeung A., Palmateer N.E., Dillon J.F., McDonald S.A., Smith S., Barclay S., Hayes P.C., Gunson R.N., Templeton K., Goldberg D.J. (2022). Population-level estimates of hepatitis C reinfection post scale-up of direct-acting antivirals among people who inject drugs. J. Hepatol..

[60] Berenguer J., Rodriguez-Castellano E., Carrero A., Von Wichmann M.A., Montero M., Galindo M.J., Mallolas J., Crespo M., Tellez M.J., Quereda C. (2017). Eradication of hepatitis C virus and non-liver-related non-acquired immune deficiency syndrome-related events in human immunodeficiency virus/hepatitis C virus coinfection. Hepatology.

[61] Diez C., Berenguer J., Ibanez-Samaniego L., Llop E., Perez-Latorre L., Catalina M.V., Hontanon V., Jimenez-Sousa M.A., Aldamiz-Echevarria T., Martinez J. (2020). Persistence of Clinically Significant Portal Hypertension After Eradication of Hepatitis C Virus in Patients With Advanced Cirrhosis. Clin. Infect. Dis..

[62] Appay V., van Lier R.A., Sallusto F., Roederer M. (2008). Phenotype and function of human T lymphocyte subsets: Consensus and issues. Cytom. A.

[63] Breen E.J., Tan W., Khan A. (2016). The Statistical Value of Raw Fluorescence Signal in Luminex xMAP Based Multiplex Immunoassays. Sci. Rep..

[64] Breen E.J., Polaskova V., Khan A. (2015). Bead-based multiplex immuno-assays for cytokines, chemokines, growth factors and other analytes: Median fluorescence intensities versus their derived absolute concentration values for statistical analysis. Cytokine.

[65] Garcia-Broncano P., Medrano L.M., Berenguer J., González-García J., Jiménez-Sousa M.Á., Carrero A., Hontañón V., Guardiola J.M., Crespo M., Quereda C. (2018). Dysregulation of the Immune System in HIV/HCV-Coinfected Patients According to Liver Stiffness Status. Cells.

[66] Wakita T., Pietschmann T., Kato T., Date T., Miyamoto M., Zhao Z., Murthy K., Habermann A., Kräusslich H.-G., Mizokami M. (2005). Production of infectious hepatitis C virus in tissue culture from a cloned viral genome. Nat. Med..

[67] Scheel T.K.H., Gottwein J.M., Jensen T.B., Prentoe J.C., Hoegh A.M., Alter H.J., Eugen-Olsen J., Bukh J. (2008). Development of JFH1-based cell culture systems for hepatitis C virus genotype 4a and evidence for cross-genotype neutralization. Proc. Natl. Acad. Sci. USA.

[68] Gottwein J.M., Scheel T.K.H., Jensen T.B., Lademann J.B., Prentoe J.C., Knudsen M.L., Hoegh A.M., Bukh J. (2009). Development and characterization of hepatitis C virus genotype 1-7 cell culture systems: Role of CD81 and scavenger receptor class B type I and effect of antiviral drugs. Hepatology.

[69] Gottwein J.M., Scheel T.K.H., Hoegh A.M., Lademann J.B., Eugen-Olsen J., Lisby G., Bukh J. (2007). Robust hepatitis C genotype 3a cell culture releasing adapted intergenotypic 3a/2a (S52/JFH1) viruses. Gastroenterology.

[70] Vigón L., Vázquez-Morón S., Berenguer J., González-García J., Jiménez-Sousa M.Á., Guardiola J.M., Crespo M., de Los Santos I., Von Wichmann M.A., Carrero A. (2019). Rapid decrease in titer and breadth of neutralizing anti-HCV antibodies in HIV/HCV-coinfected patients who achieved SVR. Sci. Rep..

[71] Rodríguez-Rodríguez M., Tello D., Yélamos B., Gómez-Gutiérrez J., Pacheco B., Ortega S., Serrano A.G., Peterson D.L., Gavilanes F. (2009). Structural properties of the ectodomain of hepatitis C virus E2 envelope protein. Virus Res..

