# Supplementary material for: Immune Factors Linked to Long-Term HCV Humoral Memory Five Years After Cure in People with HIV: A Cross-Sectional Study
Source: Pharmaceuticals (Basel). 2026 May 29;19(6):854. doi: 10.3390/ph19060854 (PMC13306004; doi:10.3390/ph19060854)
Supplement: Supplementary file 1 [file pharmaceuticals-19-00854-s001.zip › pharmaceuticals-4299867-supplementary.pdf]

Supplementary Data

Supplementary Data S1: STROBE Statement—checklist of items that should be included in reports of observational studies

	Item No	Recommendation	Page No
**Title and abstract**	1	(*a*) Indicate the study's design with a commonly used term in the title or the abstract	1, 3
		(*b*) Provide in the abstract an informative and balanced summary of what was done and what was found	3
**Introduction**			
Background/rationale	2	Explain the scientific background and rationale for the investigation being reported	4
Objectives	3	State specific objectives, including any prespecified hypotheses	4
**Methods**			
Study design	4	Present key elements of study design early in the paper	5
Setting	5	Describe the setting, locations, and relevant dates, including periods of recruitment, exposure, follow-up, and data collection	5
Participants	6	(*a*) *Cohort study*—Give the eligibility criteria, and the sources and methods of selection of participants. Describe methods of follow-up<br>*Case-control study*—Give the eligibility criteria, and the sources and methods of case ascertainment and control selection. Give the rationale for the choice of cases and controls<br>*Cross-sectional study*—Give the eligibility criteria, and the sources and methods of selection of participants	5-6
		(*b*) *Cohort study*—For matched studies, give matching criteria and number of exposed and unexposed<br>*Case-control study*—For matched studies, give matching criteria and the number of controls per case	
Variables	7	Clearly define all outcomes, exposures, predictors, potential confounders, and effect modifiers. Give diagnostic criteria, if applicable	5-7
Data sources/ measurement	8*	For each variable of interest, give sources of data and details of methods of assessment (measurement). Describe comparability of assessment methods if there is more than one group	5-7<br>SD 2-4
Bias	9	Describe any efforts to address potential sources of bias	5, 6-7, 14
Study size	10	Explain how the study size was arrived at	5-7
Quantitative variables	11	Explain how quantitative variables were handled in the analyses. If applicable, describe which groupings were chosen and why	6-7
Statistical methods	12	(*a*) Describe all statistical methods, including those used to control for confounding	6-7
		(*b*) Describe any methods used to examine subgroups and interactions	N/A
		(*c*) Explain how missing data were addressed	5
		(*d*) *Cohort study*—If applicable, explain how loss to follow-up was addressed<br>*Case-control study*—If applicable, explain how matching of cases and controls was addressed<br>*Cross-sectional study*—If applicable, describe analytical methods taking account of sampling strategy	N/A
		(*e*) Describe any sensitivity analyses	N/A
**Results**			
Participants	13*	(a) Report numbers of individuals at each stage of study—eg numbers potentially eligible, examined for eligibility, confirmed	8

		eligible, included in the study, completing follow-up, and analysed	
		(b) Give reasons for non-participation at each stage	8
		(c) Consider use of a flow diagram	
Descriptive data	14*	(a) Give characteristics of study participants (eg demographic, clinical, social) and information on exposures and potential confounders	8
		(b) Indicate number of participants with missing data for each variable of interest	8
		(c) *Cohort study*—Summarise follow-up time (eg, average and total amount)	N/A
Outcome data	15*	*Cohort study*—Report numbers of outcome events or summary measures over time	
		Case-control study—Report numbers in each exposure category, or summary measures of exposure	
		Cross-sectional study—Report numbers of outcome events or summary measures	8-9
Main results	16	(*a*) Give unadjusted estimates and, if applicable, confounder-adjusted estimates and their precision (eg, 95% confidence interval). Make clear which confounders were adjusted for and why they were included	8-9
		(*b*) Report category boundaries when continuous variables were categorized	
		(*c*) If relevant, consider translating estimates of relative risk into absolute risk for a meaningful time period	
Other analyses	17	Report other analyses done—eg analyses of subgroups and interactions, and sensitivity analyses	STs 4-6
**Discussion**			
Key results	18	Summarise key results with reference to study objectives	10
Limitations	19	Discuss limitations of the study, taking into account sources of potential bias or imprecision. Discuss both direction and magnitude of any potential bias	13-14
Interpretation	20	Give a cautious overall interpretation of results considering objectives, limitations, multiplicity of analyses, results from similar studies, and other relevant evidence	10-14
Generalisability	21	Discuss the generalisability (external validity) of the study results	14
**Other information**			
Funding	22	Give the source of funding and the role of the funders for the present study and, if applicable, for the original study on which the present article is based	17

*Give information separately for cases and controls in case-control studies and, if applicable, for exposed and unexposed groups in cohort and cross-sectional studies.

Note: An Explanation and Elaboration article discusses each checklist item and gives methodological background and published examples of transparent reporting. The STROBE checklist is best used in conjunction with this article (freely available on the Web sites of PLoS Medicine at http://www.plosmedicine.org/, Annals of Internal Medicine at http://www.annals.org/, and Epidemiology at http://www.epidem.com/). Information on the STROBE Initiative is available at www.strobe-statement.org.

Supplementary Data S2: Flow Cytometry and Gating Strategy

1. Sample Staining and Acquisition

Immunophenotyping was performed on fresh, K_2-EDTA-anticoagulated whole blood samples (100 μL) processed within maximum 24 hours of collection to preserve cellular viability and surface marker integrity [1, 2]. Samples were stained with a pre-titrated, 10-color monoclonal antibody cocktail targeting CD3, CD4, CD8, CD45RA, CD28, CD38, HLA-DR, CD57, and CD127 (see **Supplementary Table 1** for fluorochromes, clones, and manufacturers).

Incubation was carried out for 20 minutes at room temperature in the dark. Subsequently, erythrocyte lysis and leukocyte fixation were performed using the IMMUNOPREP Reagent System on a TQ-Prep™ Workstation (Beckman Coulter), following the manufacturer's standard protocol.

Data acquisition was performed on a Gallios™ flow cytometer (Beckman Coulter). Instrument performance was monitored daily using Flow-Check Pro™ Fluorospheres to ensure optical alignment and fluidic stability. A minimum of 200,000 events were collected within the lymphocyte gate for each sample to ensure statistical robustness of rare populations.

2. Gating Strategy and Subset Definition

Data analysis was conducted using Kaluza™ Analysis Software (version 1.5; Beckman Coulter). Lymphocytes were identified based on forward (FSC) and side scatter (SSC) properties. To ensure data quality, debris was excluded based on morphological properties (FSC vs. SSC), and doublets were removed using singlet gating (FSC-Area vs. FSC-Height).

- **Lineage Definition:** T-cells were defined as CD3+ and subdivided into helper (CD3+CD4+) and cytotoxic ($CD3^+CD8^+$) lineages.
- **Differentiation Status:** Within both $CD4^+$ and $CD8^+$ compartments, four distinct memory subsets were defined based on the coordinate expression of CD45RA and CD28:
    - **Naïve:** $CD45RA^+$ $CD28^+$
    - **Central Memory (CM):** $CD45RA^-$ $CD28^+$
    - **Effector Memory (EM):** $CD45RA^-$ $CD28^-$
    - **TemRA:** $CD45RA^+$ $CD28^-$
- **Functional Characterization:** The expression of functional markers was quantified as the percentage of positive cells within each specific subset. "Activated" cells were defined by the individual or co-expression of HLA-DR and CD38. "Senescent" cells were identified by CD57 expression. "Long-lived/Homeostatic" potential was assessed by the expression of the IL-7 receptor alpha chain (CD127).

3. Quality Control

Fluorescence compensation was established using single-stained controls with VersaComp Antibody Capture Beads (Beckman Coulter) and verified with stained cells. Fluorescence Minus One (FMO) controls were used to determine robust cut-off points for markers with continuous expression patterns (e.g., CD38, CD127) to ensure accurate gating and reproducibility.

Supplementary Data S3: Plasma Biomarker Quantification (Luminex Technology)

1. Sample Preparation and Quality Control

We utilized cryopreserved plasma samples stored at -80°C. Prior to the assay, samples were thawed on ice and centrifuged at 10,000 × g for 10 minutes at 4°C to remove residual particulate matter or lipid aggregates that could interfere with bead acquisition. To eliminate inter-assay variability, all samples from the cohort were analyzed in a single batch using the same lot of reagents.

2. Custom Multiplex Immunoassay Protocol

A custom-designed ProcartaPlex™ multiplex immunoassay (Bender MedSystems, Vienna, Austria) was used to simultaneously quantify 19 analytes. The panel was configured to quantify the following markers: (i) **the co-stimulatory axis**, including soluble cluster of differentiation soluble (s)CD27, sCD28, sCD80, OX40 (CD134), CD48 (B-lymphocyte activation marker/BLAST-1), inducible T-cell costimulator ligand (ICOS-L/B7-H2), B7 homolog 6 (B7-H6), and soluble glucocorticoid-induced TNF receptor-related protein (sGITR); (ii) **the inhibitory axis**, including soluble programmed death-1 (sPD-1), programmed death-ligand (PD-L)1, PD-L2, B- and T-lymphocyte attenuator (BTLA), V-domain Ig suppressor of T-cell activation (VISTA/B7-H5), T-cell immunoglobulin and mucin-domain containing-3 (TIM-3), soluble lymphocyte activation gene-3 (sLAG-3), and soluble cytotoxic T-lymphocyte-associated protein 4 (sCTLA-4); and (iii) **the inflammatory context**, including soluble tumor necrosis factor receptor I (sTNF-RI), interleukin (IL)-8, and IL-18. The assay was performed in 96-well plates according to the manufacturer's instructions. Briefly, 25 µL of clarified plasma was incubated with 50 µL of antibody-conjugated magnetic beads overnight at 4°C with constant shaking (500 rpm). After washing using an automated magnetic plate washer, detection antibodies were added and incubated for 30 minutes at room temperature, followed by a 30-minute incubation with Streptavidin-Phycoerythrin (SAPE).

3. Data Acquisition and Instrument Settings

Data were acquired on a Luminex 200™ analyzer (Luminex Corporation, Austin, TX, USA). The system was calibrated daily using the Luminex 200 Calibration and Performance Verification Kits. The following acquisition settings were applied:

- **Bead Count:** Minimum of 50 beads per analyte region.
- **Sample Volume:** 50 µL.
- **Gate Settings:** 7,500 to 15,000.
- **Timeout:** 60 seconds.

4. Data Processing and Fluorescence Intensity (FI) Justification

Raw data were analyzed using the Bio-Plex® software. In this study, we used mean fluorescence intensity (MFI) values for all statistical analyses instead of extrapolated concentrations [3]. While standard curves were included in the assay run, they were utilized exclusively as internal quality controls to verify assay performance and linearity. While standard curves were included in the assay run, they were used exclusively as internal quality controls to verify assay performance and linearity. This methodological approach was selected to maximize statistical power and data integrity based on the following criteria [3, 4]:

1. **Mitigation of Left-Censoring Bias:** Standard curves often result in a significant proportion of clinical samples falling below the lower limit of quantification (LLOQ). The use of MFI circumvents the need for data exclusion or the imputation of arbitrary values (e.g., LLOQ/2), thereby preserving the full variance of the dataset.
2. **Linearity and Sensitivity:** MFI values are monotonically related to the amount of analyte bound to the beads, providing a continuous scale that is sensitive for detecting subtle differences in biomarker levels within a single study.
3. **Avoidance of Interpolation Variance**: Deriving absolute concentrations introduces mathematical variance associated with the 5-parameter logistic (5PL) curve fitting. MFI provides a direct, unmanipulated measurement of the biological signal.
4. **Internal Consistency:** As all samples were processed in a single batch on the same plate using a single reagent lot, MFI values provide a reliable metric for internal comparisons and multivariable modeling without the error propagation associated with standard curve fitting.

Supplementary Data S4: Quantification of Humoral Immune Responses

1. Cell Lines and Viral Constructs

Human hepatoma-derived Huh7.5 cells were cultured in Dulbecco's Modified Eagle Medium (DMEM) supplemented with 10% fetal bovine serum, 4 mM L-glutamine, and antibiotics (100 U/mL penicillin/streptomycin) at 37°C in a 5% CO_2 atmosphere.

For neutralization assays, we employed the cell-culture-derived HCV (HCVcc) system using the JFH1 strain (genotype 2a) [5] and a panel of JFH1-based chimeric viruses [6-8]. These chimeras express the Core-NS2 structural regions of genotypes 1a (H77), 1b (J4), 3a (S52), and 4a (ED43), matching the most prevalent genotypes in the study cohort. Viruses were produced by electroporation of in vitro transcribed RNA into Huh7.5 cells, and viral titers were determined as focus-forming units (FFU/mL).

2. Quantification of Anti-E2 Antibodies (ELISA)

Plasma levels of antibodies binding to the HCV E2 envelope glycoprotein (HCV-E2Abs) were quantified using an in-house indirect Enzyme-Linked Immunosorbent Assay (ELISA) [9].

- **Antigen Production:** Recombinant E2 ectodomains (residues 384–661) corresponding to HCV genotypes 1a, 1b, 2a, 3a, and 4a were produced using a baculovirus expression system. The proteins were modified to include a C-terminal six-histidine tag for purification [10, 11].
- **Assay Protocol:** 96-well plates (Costar) were coated overnight at 4 °C with 500 ng of Galanthus nivalis lectin (Sigma) in phosphate-buffered saline (PBS; Corning). On the following day, 40 ng of purified E2 in 2% fetal porcine serum (FPS; Gibco) in PBS were added to the wells, which were then incubated for 1h at room temperature (RT). This was followed by a 30-minute blocking step using 2% FPS-PBS. Subsequently, human plasma samples were prepared in two-fold serial dilutions (starting at 1:30) using 2% FPS-PBS, added to the plates, and incubated for 1h at RT. Detection was achieved by adding a goat anti-human IgG secondary antibody conjugated to horseradish peroxidase (GE Healthcare) for a 1h incubation at RT. The enzymatic reaction was initiated with o-phenylenediamine dihydrochloride (OPD; Sigma) substrate for 10 min and subsequently stopped by adding 3N sulfuric acid. The optical density was measured at 493nm, with final values determined after correcting for non-specific signal observed in the healthy donor plasma pool. Throughout the process, the plates were washed extensively with PBS between each individual step.
- **Cut-off Definition:** A pool of plasma from HCV-negative healthy donors was included in each run. The positivity cut-off was defined as the mean OD of negative controls plus three standard deviations.

3. HCV Microneutralization Assay (HCV-nAbs)

The neutralizing capacity of plasma antibodies against a multi-genotype panel was assessed using a validated microneutralization assay [9, 10].

- **Protocol:** For the initial setup, Huh7.5 cells were seeded at a density of 10,000 cells per well into flat-bottom 96-well tissue culture plates, which had been previously coated with 0.1% porcine skin gelatin (Sigma). These plates were then maintained overnight at 37°C. On the following day, 200 FFU of each genotype of HCVcc were pre-incubated for 1h at 37°C with human plasma samples, prepared in 1:5 serial dilutions (commencing at a 1:30 ratio), or with a negative control plasma from a healthy donor pool. These virus-plasma mixtures were subsequently applied to the Huh7.5 cell monolayers and incubated for a 72h period at 37°C.
- **Detection:** Following the three-day infection period, the cells were fixed and sequentially stained with the primary 9E10 antibody (a gift from Charles Rice, The Rockefeller University, New York, USA) (1h at RT) and a secondary goat anti-mouse IgG-HRP antibody (1h at RT). Signal development was achieved using AEC substrate for 20 min in the dark. Throughout the procedure, plates were washed thoroughly with either PBS or PBS supplemented with 0.05% Tween. Finally, the FFU counts were determined via light microscopy.
- **Calculation:** Percentage of neutralization at each antibody dilution was calculated as [1- (foci in the presence of plasma test/foci in the presence of plasma control)] × 100%

4. Data Processing and Composite Score Calculation

To generate continuous quantitative variables for statistical analysis, data from both ELISA and neutralization assays were processed as follows [9, 10]:

1. **Curve Fitting:** Titration curves for each patient against each genotype were fitted using a non-linear regression model (one-phase decay) in GraphPad Prism v9.0 (GraphPad Software, San Diego, CA, USA).

2. **AUC Calculation:** The magnitude of the antibody response was derived by calculating the Area Under the Curve (AUC) from the fitted titration plots. This method integrates both the titer and the amplitude of the response into a single continuous value, providing a more granular resolution than endpoint titers alone.
3. **Composite Score:** To assess the overall titers of the humoral response in the multivariate models, a composite score was calculated for each participant. This score represents the arithmetic mean of the AUC values obtained against the five tested genotypes (Gt1a, Gt1b, Gt2a, Gt3a, Gt4a), serving as a global indicator of anti-E2 and neutralizing titers, respectively.

Supplementary Data S5: List of Abbreviations

List of abbreviations

95% CI: 95% Confidence Interval
aAMR: Adjusted Arithmetic Mean Ratio
AUC: Area Under the Curve
cART: Combination Antiretroviral Therapy
CD: Cluster of Differentiation
CM: Central Memory
DAA: Direct-Acting Antiviral
EM: Effector Memory
FDR: False Discovery Rate
GLM: Generalized Linear Model
Gt: Genotype
HCV: Hepatitis C Virus
HCV-E2Abs: Antibodies against HCV E2 glycoprotein
HCV-nAbs: HCV-Neutralizing Antibodies
HCVcc: Cell-culture-derived Hepatitis C Virus
HIV: Human Immunodeficiency Virus
HLA-DR: Human Leukocyte Antigen-DR
ICPs: immune checkpoints
IL: Interleukin
IQR: Interquartile Range
LLPCs: Long-Lived Plasma Cells
LSM: Liver Stiffness Measurement
MFI: Mean Fluorescence Intensity
PWH: People with HIV
sB7-H6: Soluble B7 homolog 6
sBTLA: Soluble B- and T-lymphocyte attenuator
sCD27: Soluble cluster of differentiation 27
sCD28: Soluble cluster of differentiation 28
sCD48: Soluble cluster of differentiation 48 (BLAST-1)
sCD80: Soluble cluster of differentiation 80
sCTLA-4: Soluble cytotoxic T-lymphocyte-associated protein 4
sGITR: Soluble glucocorticoid-induced TNF receptor-related protein
sICOS-L: Soluble Inducible T-cell costimulator ligand
sLAG-3: Soluble lymphocyte activation gene-3
sOX40: Soluble Tumor necrosis factor receptor superfamily member 4 (CD134)
sPD-1: Soluble programmed death-1
sPD-L1: Soluble Programmed death-ligand 1
sPD-L2: Soluble Programmed death-ligand 2
sTIM-3: Soluble T-cell immunoglobulin and mucin-domain containing-3
sTNF-RI: Soluble tumor necrosis factor receptor I
SVR: Sustained Virologic Response
sVISTA: Soluble V-domain Ig suppressor of T-cell activation
TemRA: Terminally differentiated effector memory re-expressing CD45RA T-cells

References:

1. Garcia-Broncano P, Medrano LM, Berenguer J, Gonzalez-Garcia J, Jimenez-Sousa MA, Carrero A, et al. **Dysregulation of the Immune System in HIV/HCV-Coinfected Patients According to Liver Stiffness Status**. *Cells* 2018; 7(11).
2. Garcia-Broncano P, Medrano LM, Berenguer J, Brochado-Kith O, Gonzalez-Garcia J, Jimenez-Sousa MA, et al. **Mild profile improvement of immune biomarkers in HIV/HCV-coinfected patients who removed hepatitis C after HCV treatment: A prospective study**. *J Infect* 2020; 80(1):99-110.
3. Breen EJ, Tan W, Khan A. **The Statistical Value of Raw Fluorescence Signal in Luminex xMAP Based Multiplex Immunoassays**. *Sci Rep* 2016; 6:26996.
4. Breen EJ, Polaskova V, Khan A. **Bead-based multiplex immuno-assays for cytokines, chemokines, growth factors and other analytes: median fluorescence intensities versus their derived absolute concentration values for statistical analysis**. *Cytokine* 2015; 71(2):188-198.
5. Wakita T, Pietschmann T, Kato T, Date T, Miyamoto M, Zhao Z, et al. **Production of infectious hepatitis C virus in tissue culture from a cloned viral genome**. *Nat Med* 2005; 11(7):791-796.
6. Scheel TK, Gottwein JM, Jensen TB, Prentoe JC, Hoegh AM, Alter HJ, et al. **Development of JFH1-based cell culture systems for hepatitis C virus genotype 4a and evidence for cross-genotype neutralization**. *Proc Natl Acad Sci U S A* 2008; 105(3):997-1002.
7. Gottwein JM, Scheel TK, Jensen TB, Lademann JB, Prentoe JC, Knudsen ML, et al. **Development and characterization of hepatitis C virus genotype 1-7 cell culture systems: role of CD81 and scavenger receptor class B type I and effect of antiviral drugs**. *Hepatology* 2009; 49(2):364-377.
8. Gottwein JM, Scheel TK, Hoegh AM, Lademann JB, Eugen-Olsen J, Lisby G, et al. **Robust hepatitis C genotype 3a cell culture releasing adapted intergenotypic 3a/2a (S52/JFH1) viruses**. *Gastroenterology* 2007; 133(5):1614-1626.
9. Vigon L, Vazquez-Moron S, Berenguer J, Gonzalez-Garcia J, Jimenez-Sousa MA, Guardiola JM, et al. **Rapid decrease in titer and breadth of neutralizing anti-HCV antibodies in HIV/HCV-coinfected patients who achieved SVR**. *Sci Rep* 2019; 9(1):12163.
10. Sepulveda-Crespo D, Yelamos MB, Diez C, Gomez J, Hontanon V, Torresano-Felipe F, et al. **Negative impact of HIV infection on broad-spectrum anti-HCV neutralizing antibody titers in HCV-infected patients with advanced HCV-related cirrhosis**. *Biomed Pharmacother* 2022; 150:113024.
11. Rodriguez-Rodriguez M, Tello D, Yelamos B, Gomez-Gutierrez J, Pacheco B, Ortega S, et al. **Structural properties of the ectodomain of hepatitis C virus E2 envelope protein**. *Virus Res* 2009; 139(1):91-99.

Supplementary Tables

Supplementary Table S1. List of monoclonal antibodies used for flow cytometry immunophenotyping

Marker	Fluorochrome	Clone	Manufacturer
CD3	Pacific Orange (PO)	UCHT1	Invitrogen
CD4	APC-Cyanine 7 (APC-Cy7)	OKT4	BioLegend
CD8	Pacific Blue (PB)	SK1	BioLegend
CD45RA	Phycoerythrin-Texas Red (ECD)	2H4LDH11LDB9	Beckman Coulter
CD28	Phycoerythrin (PE)	CD28.2	Beckman Coulter
CD38	APC-Cyanine 5 (APC-Cy5)	HIT2	BioLegend
HLA-DR	Allophycocyanin (APC)	L243	BioLegend
CD57	Fluorescein (FITC)	HCD57	BioLegend
CD127	Phycoerythrin-Cyanine 7 (PC7)	R34.34	Beckman Coulter

Supplementary Table S2. Phenotypic definitions and functional markers of analyzed T-cell subsets.

Cell Compartment	Subpopulation	Phenotypic Definition*	Biomarkers Analyzed
**$CD4^+$ T Cells**	**Total $CD4^+$**	$CD3^+$ $CD4^+$	CD38, HLA-DR, $CD38^+$HLA-DR^+, CD127, CD57
	**Naive**	$CD45RA^+$ $CD28^+$	CD38, HLA-DR, $CD38^+$HLA-DR^+, CD127
	**Central Memory (CM)**	$CD45RA^-$ $CD28^+$	CD38, HLA-DR, $CD38^+$HLA-DR^+, CD127, CD57
	**Effector Memory (EM)**	$CD45RA^-$ $CD28^-$	CD38, HLA-DR, $CD38^+$HLA-DR^+, CD127, CD57
	**TemRA**	$CD45RA^+$ $CD28^-$	CD38, HLA-DR, $CD38^+$HLA-DR^+, CD127, CD57
**$CD8^+$ T Cells**	**Total $CD8^+$**	$CD3^+$ $CD8^+$	CD38, HLA-DR, $CD38^+$HLA-DR^+, CD127, CD57
	**Naive**	$CD45RA^+$ $CD28^+$	CD38, HLA-DR, $CD38^+$HLA-DR^+, CD127
	**Central Memory (CM)**	$CD45RA^-$ $CD28^+$	CD38, HLA-DR, $CD38^+$HLA-DR^+, CD127, CD57
	**Effector Memory (EM)**	$CD45RA^-$ $CD28^-$	CD38, HLA-DR, $CD38^+$HLA-DR^+, CD127, CD57
	**TemRA**	$CD45RA^+$ $CD28^-$	CD38, HLA-DR, $CD38^+$HLA-DR^+, CD127, CD57

Note: Phenotypic definitions based on CD45RA and CD28 expression patterns. HLA-DR: Human Leukocyte Antigen – DR isotype. TemRA: Terminally Differentiated Effector Memory re-expressing CD45RA ($CD45RA^+$ $CD28^-$).

Supplementary Table S3. Pairwise correlation analysis of antibody responses across the five tested HCV genotypes and their composite score.

Assay / Comparison	Variable 1	Variable 2	Spearman's (ρ)	*p*-value	FDR (*q*-value)
**A. HCV-E2Abs**					
Inter-genotype correlations	Gt1a	Gt1b	0.395	**0.001**	**0.001**
	Gt1a	Gt2a	0.445	**<0.001**	**<0.001**
	Gt1a	Gt3a	0.389	**0.002**	**0.002**
	Gt1a	Gt4a	0.513	**<0.001**	**<0.001**
	Gt1b	Gt2a	0.509	**<0.001**	**<0.001**
	Gt1b	Gt3a	0.412	**0.001**	**0.001**
	Gt1b	Gt4a	0.590	**<0.001**	**<0.001**
	Gt2a	Gt3a	0.398	**0.001**	**0.001**
	Gt2a	Gt4a	0.686	**<0.001**	**<0.001**
	Gt3a	Gt4a	0.422	**0.001**	**0.001**
Correlation with Composite Score	Gt1a	Composite	0.711	**<0.001**	**<0.001**
	Gt1b	Composite	0.663	**<0.001**	**<0.001**
	Gt2a	Composite	0.849	**<0.001**	**<0.001**
	Gt3a	Composite	0.647	**<0.001**	**<0.001**
	Gt4a	Composite	0.820	**<0.001**	**<0.001**
**B. HCV-nAbs**					
Inter-genotype correlations	Gt1a	Gt1b	0.267	**0.033**	**0.045**
	Gt1a	Gt2a	0.321	**0.010**	**0.015**
	Gt1a	Gt3a	0.063	0.624	0.624
	Gt1a	Gt4a	0.536	**<0.001**	**<0.001**
	Gt1b	Gt2a	0.483	**<0.001**	**<0.001**
	Gt1b	Gt3a	0.216	0.087	0.109
	Gt1b	Gt4a	0.425	**0.001**	**0.001**
	Gt2a	Gt3a	0.090	0.481	0.515
	Gt2a	Gt4a	0.334	**0.007**	**0.013**
	Gt3a	Gt4a	0.178	0.160	0.185
Correlation with Composite Score	Gt1a	Composite	0.599	**<0.001**	**<0.001**
	Gt1b	Composite	0.646	**<0.001**	**<0.001**
	Gt2a	Composite	0.696	**<0.001**	**<0.001**
	Gt3a	Composite	0.325	**0.009**	**0.015**
	Gt4a	Composite	0.635	**<0.001**	**<0.001**

Statistics: Values represent Spearman's rank correlation coefficients (ρ) evaluating the relationship between the Area Under the Curve (AUC) of each individual tested genotype and the final composite score (arithmetic mean of the 5 genotypes). False Discovery Rate (FDR) q-values were calculated using the Benjamini-Hochberg procedure within each assay block. Values in bold indicate statistically significant associations (FDR-corrected q-value < 0.10).

Abbreviations: FDR, False Discovery Rate; Gt, Genotype; HCV, Hepatitis C Virus; HCV-E2Abs, Antibodies against HCV E2 glycoprotein; HCV-nAbs, HCV-Neutralizing Antibodies.

Supplementary Table S4. Clinical characteristics of the total cohort and analytical sub-groups.

Characteristics	All participants (N=64)	T-cell biomarkers Study (N=58)	Plasma biomarkers Study (N=50)
**Demographics**			
**Sex (male)**	51 (80%)	48 (83%)	39 (78%)
**Age (years)**	51 (48 – 54)	57.9 (54.5 – 60.4)	51 (48 – 54)
**BMI (kg/m^2)**	24.7 (21.4 – 28.7)	24.7 (21.5 – 28.8)	25.1 (21.5 – 28.8)
BMI ≥ 25 kg/m^2	31 (48%)	28 (48%)	25 (50%)
**Substance use history**			
**Smoking**			
Never	4 (6%)	3 (5%)	4 (8%)
Previously (≥ 6 months)	18 (28%)	17 (29%)	15 (30%)
Nowadays	42 (66%)	38 (66%)	31 (62%)
**High alcohol intake (>50 g/day)**	27 (42%)	25 (43%)	21 (42%)
**Intravenous drug use**			
Never	15 (23%)	13 (22%)	11 (22%)
Previously (≥ 6 months)	49 (77%)	45 (78%)	39 (78%)
**HIV-related data**			
**Prior AIDS**	2 (3%)	2 (3%)	2 (4%)
**Nadir CD4$^+$ T-cells/mm^3**	130 (84 – 214)	124.5 (83.0 – 214.0)	130 (95 – 214)
Nadir CD4$^+$ <200 cells/mm^3	47 (73%)	43 (74%)	36 (72%)
**Baseline CD4$^+$ T-cells/mm^3**	486 (282.5 – 698.0)	447.5 (278.0 – 631.0)	498.5 (280.0 – 690.0)
Baseline CD4$^+$ <500 cells/mm^3	33 (52%)	32 (55%)	25 (50%)
**Antiretroviral therapy**			
NRTI + NNRTI-based	44 (76%)	41 (71%)	33 (73%)
NRTI + PI-based	13 (22%)	13 (22%)	11 (24%)
Other	1 (2%)	4 (7%)	1 (2%)
**HCV-related data**			
**Previous IFN therapy**	34 (53%)	29 (50%)	29 (58%)
**HCV genotype**			
1	47 (73%)	42 (72%)	36 (72%)
3	9 (14%)	8 (14%)	8 (16%)
4	7 (11%)	7 (12%)	5 (10%)
Other / Mixed	1 (2%)	1 (2%)	1 (2%)
**HCV-RNA (Log$_{10}$ IU/mL)**	6.1 (5.7 – 6.6)	6.1 (5.7 – 6.5)	6.2 (5.8 – 6.7)
HCV-RNA ≥850,000 IU/mL	39 (61%)	33 (57%)	33 (66%)
**Liver disease**			
**LSM (kPa)**	21.2 (12.9 – 34.6)	21.2 (12.5 – 34.8)	21.0 (13.2 – 34.3)
<12.5 kPa	14 (22%)	14 (24%)	10 (20%)
12.5-24.9 kPa	21 (33%)	16 (28%)	18 (36%)
25-39.9 kPa	19 (30%)	19 (33%)	14 (28%)
≥40 kPa	10 (15%)	9 (15%)	8 (16%)

Statistics: Values are expressed as absolute numbers (percentages) for categorical variables and median (interquartile range [IQR]) for continuous variables.
Abbreviations: HIV, human immunodeficiency virus; HCV, hepatitis C virus; BMI, body mass index; AIDS, acquired immune deficiency syndrome; NRTI, nucleoside reverse transcriptase inhibitor; NNRTI, non-nucleoside reverse transcriptase inhibitor; PI, protease inhibitor; LSM, liver stiffness measurement.

Supplementary Table S5. Unadjusted associations between CD4+ T-cell subsets and HCV-specific antibody responses five years post-SVR (n=58).

T-cell Subset	Markers	HCV-E2Abs			HCV-nAbs		
		AMR (95% CI)	p-value	q-value	AMR (95% CI)	p-value	q-value
**Total - Base Population**	$CD38^+$	**1.37 (1.11; 1.70)**	**0.004**	**0.084**	1.94 (1.08; 3.49)	0.026	0.104
($CD4^+$)	$HLA\text{-}DR^+$	1.09 (0.92; 1.30)	0.311	0.607	1.28 (0.65; 2.49)	0.474	0.603
	$CD38^+$ $HLA\text{-}DR^+$	1.15 (1.01; 1.32)	0.031	0.145	1.25 (0.77; 2.05)	0.370	0.539
	$CD57^+$	1.16 (0.98; 1.37)	0.086	0.241	1.52 (1.00; 2.30)	0.049	0.137
	$CD127^+$	1.55 (0.67; 3.62)	0.306	0.607	2.02 (0.56; 7.27)	0.281	0.437
**Naïve CD4+**	Naïve (% total)	1.01 (0.84; 1.23)	0.880	0.948	0.79 (0.34; 1.83)	0.590	0.688
($CD4^+CD45RA^+CD28^+$)	$CD38^+$	1.20 (1.04; 1.38)	0.013	0.121	**2.38 (1.68; 3.37)**	**<0.001**	**0.028**
	$HLA\text{-}DR^+$	1.02 (0.93; 1.13)	0.641	0.855	1.21 (0.79; 1.85)	0.385	0.539
	$CD38^+$ $HLA\text{-}DR^+$	1.07 (0.99; 1.16)	0.068	0.212	1.25 (0.88; 1.78)	0.205	0.410
	$CD127^+$	**2.92 (1.35; 6.30)**	**0.006**	**0.084**	**5.25 (1.49; 18.48)**	**0.010**	**0.056**
**Central Memory (CM)**	CM	0.98 (0.63; 1.52)	0.919	0.949	0.74 (0.17; 3.27)	0.693	0.746
($CD4^+CD45RA^-CD28^+$)	$CD38^+$	1.21 (1.02; 1.42)	0.025	0.140	**2.75 (1.40; 5.40)**	**0.003**	**0.028**
	$HLA\text{-}DR^+$	1.02 (0.85; 1.23)	0.799	0.933	1.68 (0.90; 3.12)	0.103	0.240
	$CD38^+$ $HLA\text{-}DR^+$	1.06 (0.92; 1.22)	0.394	0.649	**2.16 (1.24; 3.75)**	**0.006**	**0.042**
	$CD57^+$	1.02 (0.85; 1.23)	0.800	0.933	1.71 (1.03; 2.84)	0.038	0.118
	$CD127^+$	1.03 (0.36; 2.94)	0.949	0.949	1.27 (0.07; 21.61)	0.869	0.901
**Effector Memory (EM)**	EM	1.07 (0.92; 1.25)	0.388	0.649	1.16 (0.80; 1.68)	0.430	0.573
($CD4^+CD45RA^-CD28^-$)	$CD38^+$	1.14 (0.98; 1.33)	0.095	0.242	1.12 (0.65; 1.91)	0.689	0.746
	$HLA\text{-}DR^+$	1.04 (0.87; 1.24)	0.681	0.867	0.58 (0.32; 1.05)	0.074	0.188
	$CD38^+$ $HLA\text{-}DR^+$	1.06 (0.94; 1.20)	0.325	0.607	0.73 (0.43; 1.24)	0.248	0.420
	$CD57^+$	1.14 (1.00; 1.30)	0.043	0.151	1.29 (0.94; 1.77)	0.112	0.241
	$CD127^+$	0.94 (0.77; 1.15)	0.530	0.767	0.98 (0.59; 1.61)	0.928	0.928
**TemRA**	TemRA	0.98 (0.81; 1.20)	0.858	0.948	1.21 (0.62; 2.39)	0.572	0.688
($CD4^+CD45RA^+CD28^-$)	$CD38^+$	1.19 (1.03; 1.37)	0.020	0.140	**1.67 (1.20; 2.33)**	**0.003**	**0.028**
	$HLA\text{-}DR^+$	1.09 (0.95; 1.25)	0.236	0.551	1.31 (0.82; 2.09)	0.255	0.420
	$CD38^+$ $HLA\text{-}DR^+$	1.12 (1.00; 1.25)	0.040	0.151	**1.51 (1.08; 2.12)**	**0.017**	**0.079**
	$CD57^+$	1.05 (0.91; 1.21)	0.548	0.767	1.60 (1.03; 2.47)	0.035	0.118
	$CD127^+$	1.05 (0.90; 1.22)	0.512	0.767	1.29 (0.85; 1.96)	0.229	0.420

Statistics: Data are presented as Arithmetic Mean Ratios (AMR) and 95% Confidence Intervals (95% CI) derived from unadjusted Generalized Linear Models (GLM) with a gamma distribution and log-link function. AMR represents the multiplicative effect on antibody titers per doubling (log2-increase) of the immunological marker. AMR > 1 and AMR < 1 indicate positive and inverse associations, respectively. Values in bold indicate statistically significant associations (FDR-corrected q-value < 0.10).

Abbreviations: AMR, Arithmetic Mean Ratio; 95%CI, 95% Confidence Interval; CM, Central Memory; EM, Effector Memory; FDR, False Discovery Rate; HCV, Hepatitis C Virus; nAbs, neutralizing antibodies; SVR, Sustained Virologic Response; TemRA, Terminally Differentiated Effector Memory re-expressing CD45RA.

Supplementary Table S6. Sensitivity analysis of CD4+ T-cell subsets associated HCV-E2Abs and HCV-nAbs titers five years post-SVR substituting LSM with HCV treatment regimen.

	Primary Model (LSM Adjusted)		Sensitivity Model (Treatment Adjusted)	
**Biomarker**	**aAMR (95% CI)**	**p-value**	**aAMR (95% CI)**	**p-value**
**HCV-E2Abs**				
**Total CD4⁺**				
$CD38^+$	1.58 (1.19; 2.10)	**0.002**	1.56 (1.18; 2.05)	**0.002**
$CD38^+$ $HLA\text{-}DR^+$	1.24 (1.06; 1.46)	**0.008**	1.20 (1.03; 1.41)	**0.023**
**Naïve $CD4^+$**				
$CD38^+$	1.33 (1.15; 1.54)	**<0.001**	1.22 (1.06; 1.40)	**0.005**
$CD38^+$ $HLA\text{-}DR^+$	1.13 (1.04; 1.24)	**0.006**	1.09 (1.01; 1.18)	**0.035**
$CD127^+$	2.74 (1.10; 6.80)	**0.030**	2.72 (1.16; 6.39)	**0.022**
**Central Memory (CM)**				
$CD38^+$	1.31 (1.09; 1.58)	**0.004**	1.33 (1.09; 1.63)	**0.006**
**TemRA**				
$CD38^+$	1.24 (1.07; 1.44)	**0.005**	1.22 (1.05; 1.42)	**0.010**
$CD38^+$ $HLA\text{-}DR^+$	1.15 (1.03; 1.28)	**0.013**	1.14 (1.01; 1.28)	**0.033**
**HCV-nAbs**				
**Total $CD4^+$**				
$CD38^+$	2.05 (1.14; 3.69)	**0.017**	1.55 (0.81; 2.96)	0.183
$CD57^+$	1.54 (1.06; 2.24)	**0.022**	1.26 (0.88; 1.80)	0.206
**Naïve $CD4^+$**				
$CD38^+$	2.29 (1.61; 3.25)	**<0.001**	1.95 (1.39; 2.74)	**<0.001**
$CD38^+$ $HLA\text{-}DR^+$	1.42 (1.08; 1.87)	**0.012**	1.22 (0.91; 1.63)	0.187
**Central Memory (CM)**				
$CD38^+$	2.14 (1.22; 3.75)	**0.008**	1.68 (0.90; 3.15)	0.106
$CD38^+$ $HLA\text{-}DR^+$	1.79 (1.13; 2.82)	**0.013**	1.40 (0.90; 2.19)	0.139
$HLA\text{-}DR^+$	1.70 (1.03; 2.81)	**0.039**	1.59 (1.02; 2.48)	**0.042**
**Effector Memory (EM)**				
$HLA\text{-}DR^+$	0.59 (0.36; 0.96)	**0.032**	0.71 (0.44; 1.16)	0.174
**TemRA**				
$CD127^+$	1.58 (1.22; 2.05)	**0.001**	1.19 (0.77; 1.86)	0.436
$CD57^+$	1.49 (1.05; 2.10)	**0.026**	1.32 (0.90; 1.94)	0.156
$CD38^+$	1.55 (1.05; 2.30)	**0.028**	1.39 (0.94; 2.04)	0.095

Statistics: Data are presented as adjusted Arithmetic Mean Ratios (aAMR) and 95% Confidence Intervals (95% CI) derived from Generalized Linear Models (GLM) with a gamma distribution and log-link function. The Primary Model was adjusted for age, sex, HCV genotype, nadir CD4+ T-cell count, and continuous Liver Stiffness Measurement (LSM). The Sensitivity Model substituted LSM with the historical HCV treatment regimen (IFN-based vs. DAA-based). The table displays T-cell subsets and markers that reached statistical significance ($p < 0.05$, highlighted in bold) in at least one of the models.

Abbreviations: aAMR, adjusted Arithmetic Mean Ratio; CI, Confidence Interval; CM, Central Memory; DAA, Direct-Acting Antiviral; EM, Effector Memory; HCV, Hepatitis C Virus; IFN, Interferon; LSM, Liver Stiffness Measurement; TemRA, Terminally Differentiated Effector Memory re-expressing CD45RA.

Supplementary Table S7. Unadjusted associations between CD8+ T-cell subsets and HCV-specific antibody responses five years post-SVR (n=58).

		HCV-E2Abs			HCV-nAbs		
**T-cell Subset**	**Markers**	**AMR (95% CI)**	**p-value**	**q-value**	**AMR (95% CI)**	**p-value**	**q-value**
**Total - Base Population**	CD38$^+$	1.15 (1.01; 1.30)	0.029	0.497	**1.67 (1.19; 2.34)**	**0.003**	**0.008**
(CD8$^+$)	HLA-DR$^+$	1.06 (0.90; 1.25)	0.467	0.623	**1.56 (1.07; 2.27)**	**0.020**	**0.035**
	CD38$^+$ HLA-DR$^+$	1.09 (0.96; 1.22)	0.171	0.498	**1.68 (1.19; 2.38)**	**0.004**	**0.010**
	CD57$^+$	1.18 (0.94; 1.50)	0.156	0.498	**2.20 (1.37; 3.54)**	**0.001**	**0.004**
	CD127$^+$	0.77 (0.48; 1.22)	0.262	0.498	0.38 (0.13; 1.09)	0.073	0.108
**Naïve CD8+**	Naïve (% total)	0.88 (0.75; 1.04)	0.127	0.498	**0.41 (0.20; 0.86)**	**0.019**	**0.035**
(CD8$^+$CD45RA$^+$CD28$^+$)	CD38$^+$	1.07 (0.99; 1.15)	0.070	0.497	**1.30 (1.19; 1.41)**	**<0.001**	**0.004**
	HLA-DR$^+$	1.03 (0.92; 1.16)	0.575	0.680	1.20 (0.89; 1.61)	0.241	0.281
	CD38$^+$ HLA-DR$^+$	1.06 (1.00; 1.13)	0.068	0.497	**1.28 (1.14; 1.44)**	**<0.001**	**0.004**
	CD127$^+$	1.28 (0.70; 2.36)	0.418	0.589	2.90 (0.31; 27.52)	0.353	0.380
**Central Memory (CM)**	CM	0.86 (0.67; 1.11)	0.253	0.498	0.56 (0.27; 1.18)	0.128	0.171
(CD8$^+$CD45RA$^-$CD28$^+$)	CD38$^+$	1.01 (0.88; 1.15)	0.896	0.896	1.44 (0.93; 2.23)	0.104	0.146
	HLA-DR$^+$	1.09 (0.89; 1.33)	0.421	0.589	**2.36 (1.45; 3.84)**	**<0.001**	**0.004**
	CD38$^+$ HLA-DR$^+$	1.03 (0.90; 1.17)	0.661	0.712	**1.56 (1.04; 2.34)**	**0.033**	0.051
	CD57$^+$	1.11 (0.94; 1.32)	0.217	0.498	**2.16 (1.24; 3.74)**	**0.006**	**0.013**
	CD127$^+$	1.25 (0.57; 2.72)	0.583	0.680	1.58 (0.11; 23.43)	0.738	0.738
**Effector Memory (EM)**	EM	1.10 (0.93; 1.31)	0.267	0.498	1.38 (0.82; 2.34)	0.227	0.276
(CD8$^+$CD45RA-CD28$^-$)	CD38$^+$	1.08 (0.97; 1.20)	0.182	0.498	**1.75 (1.32; 2.32)**	**<0.001**	**0.004**
	HLA-DR$^+$	1.08 (0.91; 1.28)	0.387	0.589	**1.85 (1.20; 2.85)**	**0.005**	**0.012**
	CD38$^+$ HLA-DR$^+$	1.07 (0.95; 1.20)	0.258	0.498	**1.77 (1.34; 2.34)**	**<0.001**	**0.004**
	CD57$^+$	1.23 (0.91; 1.65)	0.179	0.498	**2.18 (1.10; 4.32)**	**0.026**	**0.043**
	CD127$^+$	0.92 (0.73; 1.17)	0.514	0.654	0.74 (0.44; 1.26)	0.272	0.305
**TemRA**	TemRA	1.15 (0.91; 1.45)	0.241	0.498	1.49 (0.83; 2.69)	0.183	0.233
(CD8$^+$CD45RA$^+$CD28$^-$)	CD38$^+$	1.08 (0.99; 1.18)	0.071	0.497	**1.58 (1.30; 1.93)**	**<0.001**	**0.004**
	HLA-DR$^+$	0.99 (0.94; 1.04)	0.643	0.712	**1.18 (1.07; 1.31)**	**0.002**	**0.006**
	CD38$^+$ HLA-DR$^+$	1.01 (0.95; 1.07)	0.704	0.730	**1.26 (1.12; 1.43)**	**<0.001**	**0.004**
	CD57$^+$	1.14 (0.85; 1.52)	0.376	0.589	**2.34 (1.25; 4.39)**	**0.008**	**0.016**
	CD127$^+$	0.92 (0.75; 1.12)	0.392	0.589	0.81 (0.50; 1.32)	0.401	0.416

Statistics: Data are presented as Arithmetic Mean Ratios (AMR) and 95% Confidence Intervals (95% CI) derived from unadjusted Generalized Linear Models (GLM) with a gamma distribution and log-link function. AMR represents the multiplicative effect on antibody titers per doubling (log2-increase) of the immunological marker. AMR > 1 and AMR < 1 indicate positive and inverse associations, respectively. Values in bold indicate statistically significant associations (FDR-corrected q-value < 0.10).
Abbreviations: AMR, Arithmetic Mean Ratio; 95%CI, 95% Confidence Interval; CM, Central Memory; EM, Effector Memory; FDR, False Discovery Rate; HCV, Hepatitis C Virus; nAbs, neutralizing antibodies; TemRA, Terminally Differentiated Effector Memory re-expressing CD45RA.

Supplementary Table S8. Sensitivity analysis of CD8+ T-cell subsets associated HCV-E2Abs and HCV-nAbs titers five years post-SVR substituting LSM with HCV treatment regimen.

Biomarker	Primary Model (LSM Adjusted)		Sensitivity Model (Treatment Adjusted)	
	aAMR (95% CI)	p-value	aAMR (95% CI)	p-value
**HCV-E2Abs**				
**Total CD8$^+$**				
CD38$^+$	1.18 (1.04; 1.33)	**0.008**	1.17 (1.04; 1.32)	**0.008**
CD38$^+$ HLA-DR$^+$	1.14 (1.01; 1.29)	**0.040**	1.13 (1.00; 1.27)	0.057
**Naïve CD8$^+$**				
CD38$^+$ HLA-DR$^+$	1.08 (1.02; 1.14)	**0.013**	1.07 (1.00; 1.14)	**0.034**
CD38$^+$	1.10 (1.02; 1.19)	**0.018**	1.09 (1.00; 1.18)	**0.038**
**TemRA**				
CD38$^+$	1.10 (1.01; 1.20)	**0.035**	1.09 (1.00; 1.19)	0.053
**HCV-nAbs**				
**Total CD8$^+$**				
HLA-DR$^+$	1.59 (1.02; 2.50)	**0.042**	1.26 (0.78; 2.02)	0.341
**Naïve CD8$^+$**				
Naïve (% total)	0.41 (0.23; 0.73)	**0.003**	0.41 (0.20; 0.86)	**0.018**
CD38$^+$	1.17 (1.04; 1.32)	**0.012**	1.07 (0.93; 1.23)	0.334
**Central Memory (CM)**				
HLA-DR$^+$	2.17 (1.26; 3.76)	**0.005**	1.65 (0.92; 2.96)	0.090
**Effector Memory (EM)**				
HLA-DR$^+$	1.80 (1.17; 2.76)	**0.007**	1.56 (1.02; 2.38)	**0.042**
CD38$^+$ HLA-DR$^+$	1.53 (1.08; 2.15)	**0.016**	1.27 (0.86; 1.87)	0.229
EM (% total)	1.57 (1.08; 2.28)	**0.017**	1.43 (0.98; 2.08)	0.066
**TemRA**				
CD38$^+$	1.47 (1.19; 1.81)	**<0.001**	1.36 (1.07; 1.71)	**0.010**
CD38$^+$ HLA-DR$^+$	1.21 (1.05; 1.40)	**0.008**	1.13 (0.97; 1.32)	0.114
HLA-DR$^+$	1.16 (1.02; 1.32)	**0.025**	1.13 (1.01; 1.27)	**0.039**

Statistics: Data are presented as adjusted Arithmetic Mean Ratios (aAMR) and 95% Confidence Intervals (95% CI) derived from Generalized Linear Models (GLM) with a gamma distribution and log-link function. The Primary Model was adjusted for age, sex, HCV genotype, nadir CD4+ T-cell count, and continuous Liver Stiffness Measurement (LSM). The Sensitivity Model substituted LSM with the historical HCV treatment regimen (IFN-based vs. DAA-based). The table displays T-cell subsets and markers that reached statistical significance ($p < 0.05$, highlighted in bold) in at least one of the models.
Abbreviations: aAMR, adjusted Arithmetic Mean Ratio; CI, Confidence Interval; CM, Central Memory; DAA, Direct-Acting Antiviral; EM, Effector Memory; HCV, Hepatitis C Virus; IFN, Interferon; LSM, Liver Stiffness Measurement; TemRA, Terminally Differentiated Effector Memory re-expressing CD45RA.

Supplementary Table S9. Unadjusted associations of plasma biomarkers with HCV-E2Abs and HCV-nAbs titers five years post-SVR (n=50).

	HCV-E2Abs			HCV-nAbs		
**Biomarker (MFI)**	**AMR (95% CI)**	**p-value**	**q-value**	**AMR (95% CI)**	**p-value**	**q-value**
**A. Co-stimulatory Axis**						
sCD27	**1.44 (1.14; 1.80)**	**0.002**	**0.038**	1.16 (0.43; 3.11)	0.773	0.814
sCD28	0.97 (0.84; 1.13)	0.718	0.876	1.39 (0.90; 2.13)	0.139	0.264
sCD80	0.95 (0.81; 1.12)	0.536	0.876	1.49 (0.96; 2.31)	0.077	0.205
sOX40 (CD134)	1.23 (0.89; 1.70)	0.209	0.794	2.21 (0.77; 6.34)	0.139	0.264
sCD48 (BLAST-1)	1.00 (0.80; 1.25)	0.983	0.983	1.03 (0.42; 2.51)	0.956	0.998
sICOS-L (B7-H2)	1.19 (0.87; 1.63)	0.271	0.794	0.71 (0.33; 1.54)	0.386	0.609
sB7-H6	1.10 (0.73; 1.66)	0.642	0.876	0.35 (0.13; 0.91)	0.031	0.147
sGITR	0.94 (0.78; 1.15)	0.568	0.876	0.91 (0.51; 1.63)	0.761	0.814
**B. Inhibitory Axis**						
sPD-1	1.01 (0.85; 1.19)	0.955	0.983	1.00 (0.62; 1.61)	0.998	0.998
sPD-L1	0.95 (0.86; 1.04)	0.279	0.794	0.84 (0.34; 2.08)	0.705	0.814
sPD-L2	1.11 (0.83; 1.51)	0.479	0.876	0.99 (0.42; 2.33)	0.986	0.998
sBTLA	0.97 (0.84; 1.12)	0.686	0.876	1.12 (0.70; 1.80)	0.643	0.814
sVISTA (B7-H5)	1.01 (0.88; 1.17)	0.876	0.924	1.91 (0.82; 4.45)	0.132	0.264
sTIM-3	1.18 (0.92; 1.50)	0.195	0.794	1.32 (0.57; 3.01)	0.517	0.689
sLAG-3	1.04 (0.84; 1.28)	0.723	0.876	1.58 (0.83; 3.02)	0.164	0.312
sCTLA-4	0.99 (0.86; 1.14)	0.886	0.924	1.26 (0.96; 1.66)	0.093	0.219
**C. Inflammatory Context**						
sTNF-RI	1.09 (0.97; 1.24)	0.157	0.794	1.38 (0.98; 1.95)	0.064	0.205
IL-8	1.14 (0.96; 1.34)	0.137	0.794	1.65 (0.91; 2.99)	0.098	0.219
IL-18	0.98 (0.87; 1.10)	0.730	0.876	**0.60 (0.42; 0.84)**	**0.003**	**0.057**

Statistics: Data are presented as Arithmetic Mean Ratios (AMR) and 95% Confidence Intervals (95% CI). Values were derived from Generalized Linear Models (GLM) with a gamma distribution and log-link function. AMR represents the multiplicative effect on antibody outcomes per doubling (log2-increase) of the biomarker level. Values in bold indicate statistically significant associations (FDR-corrected q-value < 0.10).

Abbreviations: AMR, Arithmetic Mean Ratio; sB7-H6, soluble Natural cytotoxicity triggering receptor 3 ligand 1; sBTLA, soluble B- and T-lymphocyte attenuator; CI, Confidence Interval; FDR, False Discovery Rate; HCV, Hepatitis C Virus; sICOS-L, soluble Inducible T-cell costimulator ligand; IL, Interleukin; LSM, Liver Stiffness Measurement; nAbs, neutralizing antibodies; sOX40 (CD134), soluble Tumor necrosis factor receptor superfamily member 4; sPD-L1, soluble Programmed death-ligand 1; sPD-L2, soluble Programmed death-ligand 2; sCD27, Soluble Cluster of Differentiation 27; sTNF-RI, Soluble Tumor Necrosis Factor Receptor I; SVR, Sustained Virological Response; sTIM-3, soluble T-cell immunoglobulin and mucin-domain containing-3; sVISTA, soluble V-domain Ig suppressor of T cell activation; MFI, mean fluorescence intensity.

Supplementary Table S10. Sensitivity analysis of plasma biomarkers associated HCV-E2Abs and HCV-nAbs titers five years post-SVR substituting LSM with HCV treatment regimen.

	Primary Model (LSM Adjusted)		Sensitivity Model (Treatment Adjusted)	
**Biomarker**	**aAMR (95% CI)**	**p-value**	**aAMR (95% CI)**	**p-value**
**HCV-E2Abs**				
sCD27	1.46 (1.15; 1.85)	**0.002**	1.53 (1.21; 1.92)	**<0.001**
sICOS-L (B7-H2)	1.27 (0.83; 1.95)	0.272	1.59 (1.04; 2.45)	**0.033**
**HCV-nAbs**				
sCD27	2.11 (0.84; 5.32)	0.112	2.92 (1.37; 6.22)	**0.006**
sCD28	1.68 (1.19; 2.38)	**0.003**	1.69 (1.18; 2.41)	**0.004**
sCD80	1.48 (1.01; 2.17)	**0.045**	1.41 (1.00; 1.98)	**0.049**
sOX40 (CD134)	2.25 (0.71; 7.14)	0.169	2.79 (1.15; 6.76)	**0.023**
sICOS-L (B7-H2)	1.88 (0.80; 4.43)	0.147	2.37 (1.10; 5.10)	**0.027**
sGITR	1.55 (1.01; 2.38)	**0.047**	1.37 (0.85; 2.20)	0.202
sPD-L2	2.50 (1.29; 4.82)	**0.006**	4.13 (1.89; 9.02)	**<0.001**
sTIM-3	1.98 (1.00; 3.89)	**0.049**	2.28 (1.04; 5.00)	**0.039**
sLAG-3	1.97 (1.17; 3.30)	**0.010**	2.03 (1.30; 3.19)	**0.002**
sCTLA-4	1.46 (1.07; 1.99)	**0.018**	1.42 (1.07; 1.87)	**0.015**
sTNF-RI	1.44 (1.16; 1.79)	**0.001**	1.30 (0.98; 1.74)	0.074
IL-18	0.53 (0.36; 0.78)	**0.001**	0.57 (0.40; 0.83)	**0.003**

Statistics: Data are presented as adjusted Arithmetic Mean Ratios (aAMR) and 95% Confidence Intervals (95% CI) derived from Generalized Linear Models (GLM) with a gamma distribution and log-link function. The Primary Model was adjusted for age, sex, HCV genotype, nadir CD4+ T-cell count, and continuous Liver Stiffness Measurement (LSM). The Sensitivity Model substituted LSM with the historical HCV treatment regimen (IFN-based vs. DAA-based) to assess robustness against structural collinearity. The table displays biomarkers that reached statistical significance ($p < 0.05$, highlighted in bold) in at least one of the models.

Abbreviations: aAMR, adjusted Arithmetic Mean Ratio; CI, Confidence Interval; DAA, Direct-Acting Antiviral; HCV, Hepatitis C Virus; IFN, Interferon; LSM, Liver Stiffness Measurement.
